# Phylogenetic analysis and stress response of the plant U2 small nuclear ribonucleoprotein B″ gene family

**DOI:** 10.1186/s12864-022-08956-0

**Published:** 2022-11-08

**Authors:** Cong Gao, Shuai Lu, Rong Zhou, Junjie Ding, Jialiang Fan, Binying Han, Moxian Chen, Baohua Wang, Yunying Cao

**Affiliations:** 1 School of Life Sciences, Nantong University, Nantong, 226019 Jiangsu China; 2 National Key Laboratory of Plant Molecular Genetics and CAS Center for Excellence in Molecular Plant Sciences, Institute of Plant Physiology & Ecology, Chinese Academy of Sciences, Shanghai, 200032 China; 3 CAS Key Laboratory of Quantitative Engineering Biology, Shenzhen Institute of Synthetic Biology, Shenzhen Institutes of Advanced Technology, Chinese Academy of Sciences, Shenzhen, 518055 China

**Keywords:** Splicing factor, U2B″, Bioinformatics, Subcellular localisation

## Abstract

**Background:**

Alternative splicing (AS) is an important channel for gene expression regulation and protein diversification, in addition to a major reason for the considerable differences in the number of genes and proteins in eukaryotes. In plants, U2 small nuclear ribonucleoprotein B″ (U2B″), a component of splicing complex U2 snRNP, plays an important role in AS. Currently, few studies have investigated plant U2B″, and its mechanism remains unclear.

**Result:**

Phylogenetic analysis, including gene and protein structures, revealed that U2B″ is highly conserved in plants and typically contains two RNA recognition motifs. Subcellular localisation showed that OsU2B″ is located in the nucleus and cytoplasm, indicating that it has broad functions throughout the cell. Elemental analysis of the promoter region showed that it responded to numerous external stimuli, including hormones, stress, and light. Subsequent qPCR experiments examining response to stress (cold, salt, drought, and heavy metal cadmium) corroborated the findings. The prediction results of protein–protein interactions showed that its function is largely through a single pathway, mainly through interaction with snRNP proteins.

**Conclusion:**

U2B″ is highly conserved in the plant kingdom, functions in the nucleus and cytoplasm, and participates in a wide range of processes in plant growth and development.

**Supplementary Information:**

The online version contains supplementary material available at 10.1186/s12864-022-08956-0.

## Background

In contrast to the model of the 1 gene-1 polypeptide in prokaryotes, eukaryotic genes contain exons and introns. They generate mature mRNAs via splicing, which is an important post-transcriptional regulatory link during pre-mRNA splicing [1]. In the splicing process, the U1 snRNP identifies and binds to the 5'-terminal splicing site of the intron [2]. Subsequently, U2 cofactor identifies the 3' terminal splicing site to make the U2 snRNP combine with the branching point and form a pre-spliceosome (complex A). Afterwards, U4/U6 and U5 snRNPs bind to complex A and form complex B. Subsequently, the U4/U6 snRNP dissociates, and the U2 snRNP combines with the U6 snRNP to form a catalytic centre [3, 4]. Shortly afterwards, U1 and U4 snRNPs are removed from the complex, and U6 snRNPs bind to the 5' terminal splicing site to form a complex C-spliceosome [5]. Finally, a splicing reaction occurs. Different transcripts are produced by alternative splicing (AS) and are eventually translated into different proteins [6]. In eukaryotes, AS is an important channel of protein diversification and gene expression regulation and includes five modes. In addition, polyadenylation sites and variable selection of promoters can increase mRNA diversity [7]. Splicing is regulated by trans-acting factors, splicing regulatory elements (SREs) and RNA secondary structures [8]. SREs can be classified into silencers and enhancers. Silencers include intron splicing silencers (ISSs) and exon splicing silencers (ESSs), and enhancers include intron splicing enhancers (ISEs) and exon splicing enhancers (ESEs) [9]. Most activating proteins that bind to ISEs and ESEs are splicing regulatory (SR) proteins, including one or two RNA recognition motifs (RRMs) and one SR domain containing abundant arginine and serine. RRM recognised by two amino acid sequences (RNP1 and RNP2) is the most commonly used RNA-binding domain in eukaryotes [10]. A typical view of RNA–RRM interactions is that single-stranded RNA binds to a β-folded surface. Good electrostatic interactions, hydrogen bonding, and stacking between RNA bases and aromatic residues located in the RNP motif are considered major factors involved in RNA binding [11]. Splicing is also regulated by the trans-acting factor-spliceosome, which includes five small nuclear RNAs and more than 100 core proteins [12].

Spliceosomes can be divided into two categories: major and minor spliceosomes [13]. Major spliceosomes, including U1, U2, U4, U5, and U6 snRNPs [14], remove introns whose 5' termini have a “GU” splice site and whose 3' termini have an “AG” splicing site. Minor spliceosomes [15], including U5, U11, U12, U4, and U6 [16], are splice introns whose 5' termini have an “AU” splice site and whose 3' termini have an “AC” splicing site. The spliceosome is a multi-subunit RNA–protein complex. Currently, the identified splicing-related proteins are divided into five types: major snRNP proteins, splicing factors, splicing regulation factors, novel spliceosome proteins, and possible splicing-related proteins. Major snRNP proteins are divided into seven protein families: Sm core proteins, U1 snRNP-specific proteins, 17S U2 snRNP-specific proteins, U5 snRNP-specific proteins, U4/U6 snRNP-specific proteins, Tri-snRNP-specific proteins, and 18S U11/U2 snRNP-specific proteins. The major types of spliceosome-U2-dependent proteins contain several guanosine-rich U-snRNPs (U1, U2, U5, and U4/U6) and an array of non-snRNP proteins. U4 and U6 typically form complexes. Each snRNP type [17], including a conserved U-snRNA, binds to the ring containing seven Sm core proteins (B/B″, D3, D2, D1, E, F, and G) and a specific protein of each snRNP. Sm proteins are highly conserved RNA-binding proteins classified into seven Sm core proteins and like-Sm (LSm) proteins. The heptamer ring formed by the seven core Sm proteins is the basic structure that binds most snRNPs. Only U6 snRNP binds to LSm2-8 to form heptamers [18].

Recent research has shown that the structure of human 17S U2 snRNP is consistent with the electron microscope structure of previously isolated U2 snRNP [19, 20] and its entire structure in the human spliceosome [21–24]. Human 17S U2 snRNP presents a bipartite three-dimensional (3D) structure, as observed through high-resolution cryo-electron microscopy. The 17S U2 snRNP is located in the nucleus and is the active form of the U2 snRNP. It binds to the branching point of the pre-mRNA during the splicing assembly. The U2 snRNP plays an important role in selecting the mRNA precursor branching site adenosine, a nucleophile, in the first step of splicing [25]. The mature 17S U2 snRNP includes 12S U2 snRNP formed by U2 snRNA, Sm protein, U2A', and U2B″ [26], and two splicing factors (SF3a and SF3b) [27]. The first step in 17S U2 snRNP formation involves the Sm protein complex and U2A'-U2B″ dimer binding to the Sm site and stem-loop IV of U2 snRNA, respectively, forming 12S U2 (Fig. S1). In addition, the binding of U2B″ to the corresponding sites requires the participation of U2A', and the specific binding of U2B″ may be attributed to this common binding. In vitro, U2B″ is able to bind human stem-loop IV, Drosophila U2 snRNA stem-loop IV, and stem-loop II of human U1 snRNA. Yeast U2 snRNA nucleotides that form base pairs with branching sites were initially isolated in a fulcrum-interacting stem-loop [28]; however, it is not clear whether human U2 snRNA folds in a similar manner. The second step is to combine SF3b with 12S U2 to form 15S U2 snRNP and then combine with SF3a to form functional 17S U2 snRNP [29]. As a spliceosomal protein, U2B″, which is an important tool for studying the evolution of RNA-binding patterns and RNA-binding specificity, belongs to the U1A/U2B″/SNF family. Although a high degree of sequence and structural conservation has been found in the family [30], proteins from modern members of this family have unique RNA-binding properties [31]. Three proteins recognise their RNA targets using RNA recognition motifs (RRMs); however, their N-terminal RRMs differ from most RRMs in their high affinity and specificity for RNA. Notably, these homologous proteins contain two RRMs, whereas yeast contains only one [32]. These phenomena were probably caused by evolution. The RRMs of the three proteins showed a high degree of conservation, similar to the two RNA stem loops that they recognised. When comparing modern RRMs, it has become apparent that each has unique RNA-binding properties, while U1A binds exclusively to U1 stem-loop II and U2B″ binds exclusively to U2 stem-loop IV in humans [33]. Although they are components of snRNPs, how U1A and U2B″ participate in splicing remains unclear; in fact, snRNP recombination had no effect on splicing in the absence of U1A in vitro [34]. Other experiments used fly homologous SNF mutations to exclude the protein from either U1 or U2 snRNP, resulting in relatively mild phenotypic consequences [35]. In contrast, mutants that simultaneously knock out U1A and U2B″ in *C. elegans* are embryonically lethal, similar to knocking out SNF in *Drosophila* [36]. It is possible that these proteins have other functions. In worms, U1A and U2B″, which are functionally redundant, interact with various snRNPs. The knockout of these two genes is necessary for a lethal phenotype. However, the absence of one does not lead to a lethal phenotype, and the other replaces the missing phenotype and binds to its corresponding snRNP. U2B″ homologous genes have been identified in vertebrates, yeast, and plants. Although the genes play an important role in the formation of the spliceosome in yeast, their mechanisms of action remain unclear. Analysis of the *C. elegans* genome has revealed two members of the U1A/U2B″ family. Notably, the two genes are present in a single operon. None of them has unique sequence features [32]. The phenomenon is difficult to explain from a molecular perspective, especially based on protein structure. Therefore, analysing the function of proteins from an evolutionary perspective could provide a novel avenue for enhancing our understanding protein function.

Especially in plants, little is known about the specific functions of U2B″. Consequently, there is a growing interest in the elucidation of plant U2B″ proteins and how they mediate U2 snRNP, which could also involve numerous U2B″-interacting proteins. The present study provides a phylogenetic description of plant U2B″ and provides a clue for future functional studies in plant AS regulation. In this work, we studied the U2B″ gene family across different plants and analysed their phylogenetics, gene and protein structures, spatiotemporal gene expression profiles under different stimulation conditions, and subcellular localisation. The results of the present study will provide basic information on the phylogeny, structure, and expression of the gene family and lay a foundation for further functional identification of plants. The design process for the entire study is illustrated in Fig. S2.

## Results

### Phylogenetic analysis of plant U2B″ gene family

To identify U2B″ genes in different plant species, we conducted a BLAST search using the *Arabidopsis* U2B″ protein (*AT2G30260*) against the Phytozome database. After filtering the sequences, 117 putative U2B″ sequences from 80 different plant species were obtained. The sequences were divided into five subfamilies (Fig. S3), including 45 dicotyledons and two monocotyledons in blue, 17 monocotyledons in pink, one fern in white, four bryophytes in green, and nine algae in yellow. Regarding the distribution of the number of genes in the 80 species, 50 species contained one gene, 24 species contained two genes, five species contained three genes (*Gossypium hirsutum, Malus domestica, Medicago truncatula, Populus trichocarpa, Salix purpurea*), and one species (*Triticum aestivum*) contained four genes (Table S1).

Gene copy numbers and species were mapped to a phylogenetic tree (Fig. S3). There were 45 dicotyledons, including 23 species with a single copy of the gene, 17 species with a double copy, and five species with three copies, and two monocotyledons, including one species with a single copy and one species with a double copy in the blue subfamily. In addition, the pink subfamily contained only monocotyledons, with a total of 17 species, including 12 species with a single copy, four species with a double copy, and one species with four copies. The white subfamily contained only one fern with a single copy. The green subfamily includes three bryophytes and three monocotyledons. Monocotyledons were all single copies, whereas bryophytes included two species with single copies and one species with a double copy. The yellow subfamily consisted of nine algae and contained eight species with single copies and one species with a double copy.

The 117 sequences from 80 plant species could provide us with a more comprehensive view of the evolutionary and developmental relationships among U2B″gene family. The clear topological structure and high bootstrap values (red) supporting each branch indicate the validity of this phylogenetic reconstruction of the U2B″ gene family. In addition, the yellow subfamily constitutes the basic part of the phylogenetic tree, which is far from other subfamilies, indicating that it is significantly associated with U2B″ in other plants. The green subfamily represents bryophytes, and the white subfamily of ferns is closely related to the yellow subfamily, which represents algae. However, the pink subfamily, representing monocotyledons, is closely related to the blue subfamily, representing dicotyledons, and is located far from the three subfamilies mentioned above. Multiple sequences from the same species were closely clustered in the same subfamily. Notably, each subfamily contained not only the plants it represented but also plants represented by other subfamilies. For example, the green subfamily contained not only bryophytes but also monocotyledons (*Ananas comosus, Spirodela polyrhiza, Zostera marina*). The blue subfamily, which represents dicotyledons, also contained two monocotyledons (*Ananas comosus, Musa acuminata*). The phenomena may be attributed to the degrees of plant development and evolution. Excluding the example above, the subfamilies strictly corresponded to the plants they represented.

## Analysis of gene structure

**Fig. 1:**
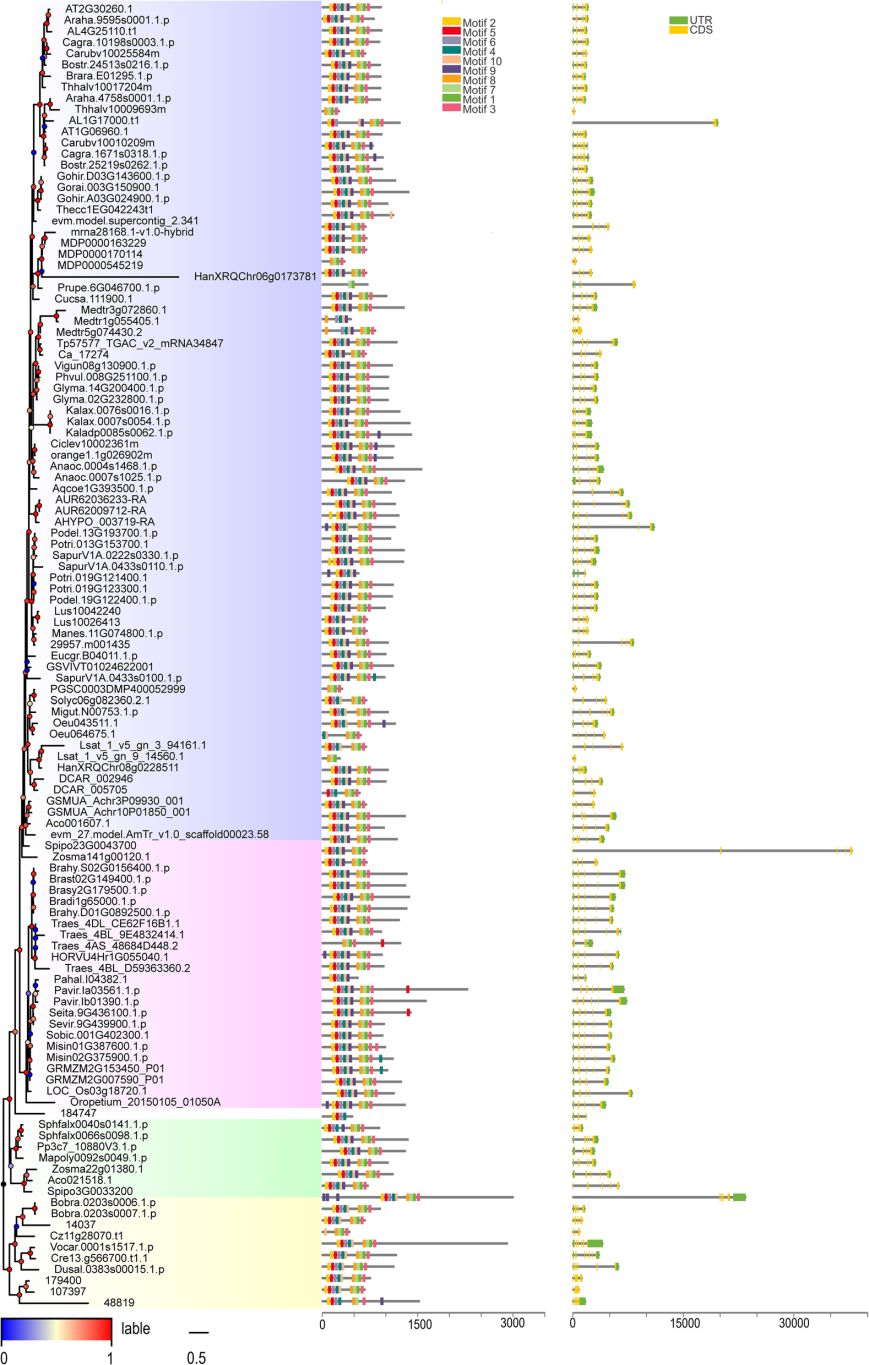
Genomic structure organisation and identification of conserved DNA motifs among plant *U2B″*. Gene structure (right panel) and identified conserved motifs (middle panel) in cDNA (by MEME analysis) are shown against the vertical phylogenetic tree (left panel). The x-axis represents the length of the gene sequence. The black vertical lines in phylogenetic analysis represent a break at that particular branch. Blue for dicotyledons, pink for monocotyledons, white for ferns, green for bryophytes, and yellow for algae

To understand the general function of plant U2B″, it is necessary to study its genomic structure and its conserved gene motifs. For visualisation, the gene structure and corresponding motifs were linked in a phylogenetic tree (Fig. 1). Surprisingly, more than 80 sequences showed a 5 exon-2 UTR structure (Fig. 1, right panel). The proportion was close to 70%, indicating that they are highly conserved in the plant genomic structure across plant genomes, which suggests strong functional conservation of U2B″. Most of the remaining sequences had 3–6 exons, with a few exceptions. For example, *Chlamydomonas* contains seven exons. It should be noted that there were a few sequences with a single intron, or even no introns. For example, one of the two sequences of *Arabidopsis thaliana* had two introns, and the other had only one intron, whereas one of the two sequences of *Capsella rubella* had two introns and the other had no introns. In addition, the gene structures of the yellow, green, and white subfamilies were smaller, the pink subfamily was larger, and the blue subfamily was different in size, and there was no obvious trend.

Although gene structure variation was minimal among U2B″, we determined whether motif composition in their cDNA sequences reflected any differences. Further investigation of conserved DNA motifs showed that 89% of the 104 sequences had similar sequence characteristics and contained 8–10 of the top 10 identified motifs (Fig. S4A) and were located at similar sequence positions (Fig. 1, middle panel). The remaining 13 sequences, whose motifs were less than eight in number, were evenly distributed in all subfamilies except the green subfamily. Therefore, the gene structure may have a subtle relationship with conserved motifs.

## Analysis of protein structure

**Fig. 2:**
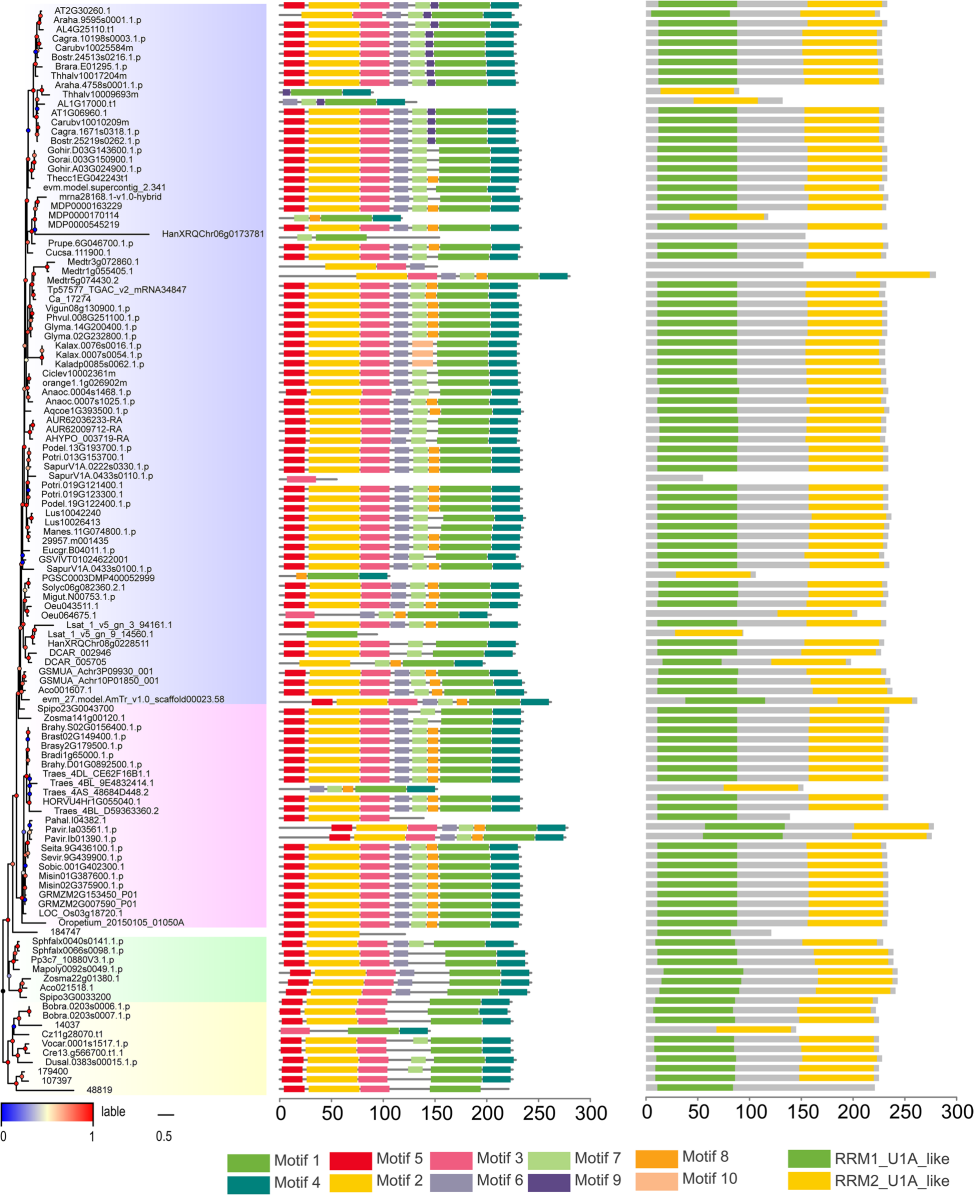
Protein structure organisation and identification of conserved amino-acid motifs among plant U2B″. Protein structure (right panel) and identified conserved amino acid motifs (middle panel) in the protein sequences with MEME analysis are shown against the vertical phylogenetic tree (left panel). The x-axis represents the length of the protein sequence. The black vertical lines in the phylogenetic analysis represent break at that particular branch. Blue for dicotyledons, pink for monocotyledons, white for ferns, green for bryophytes, and yellow for algae

Conserved domains and motifs were analysed for phylogenetic tree construction (Fig. 2). Different subfamilies of proteins were highly conserved (Fig. S5). A total of 106 peptides were predicted to contain an N-terminal domain named RRM1 and C-terminal domain named RRM2 (Fig. 2, right panel). In addition, there were eight peptides with only one RRM2, and one peptide with only one RRM1. This may lead to a decline in RNA recognition capacity. It is worth noting that two peptides (*Prunus persica, Salix purpurea*) did not have RRMs, possibly because they had more than one copy of the gene. Although some domains are lost during development and evolution, other peptides can perform similar functions.

Since the differences in protein domains were very minor, we attempted to explore differences in the motifs (Fig. 2, middle panel) of the protein sequences, with obvious differences identified. The numbers of motifs in the yellow, green, and white subfamilies were lower than those in the pink and blue subfamilies. The differences could be related to the different degrees of development and evolution among different subfamilies.

### Homology modelling and amino acid conservation estimation

**Fig. 3:**
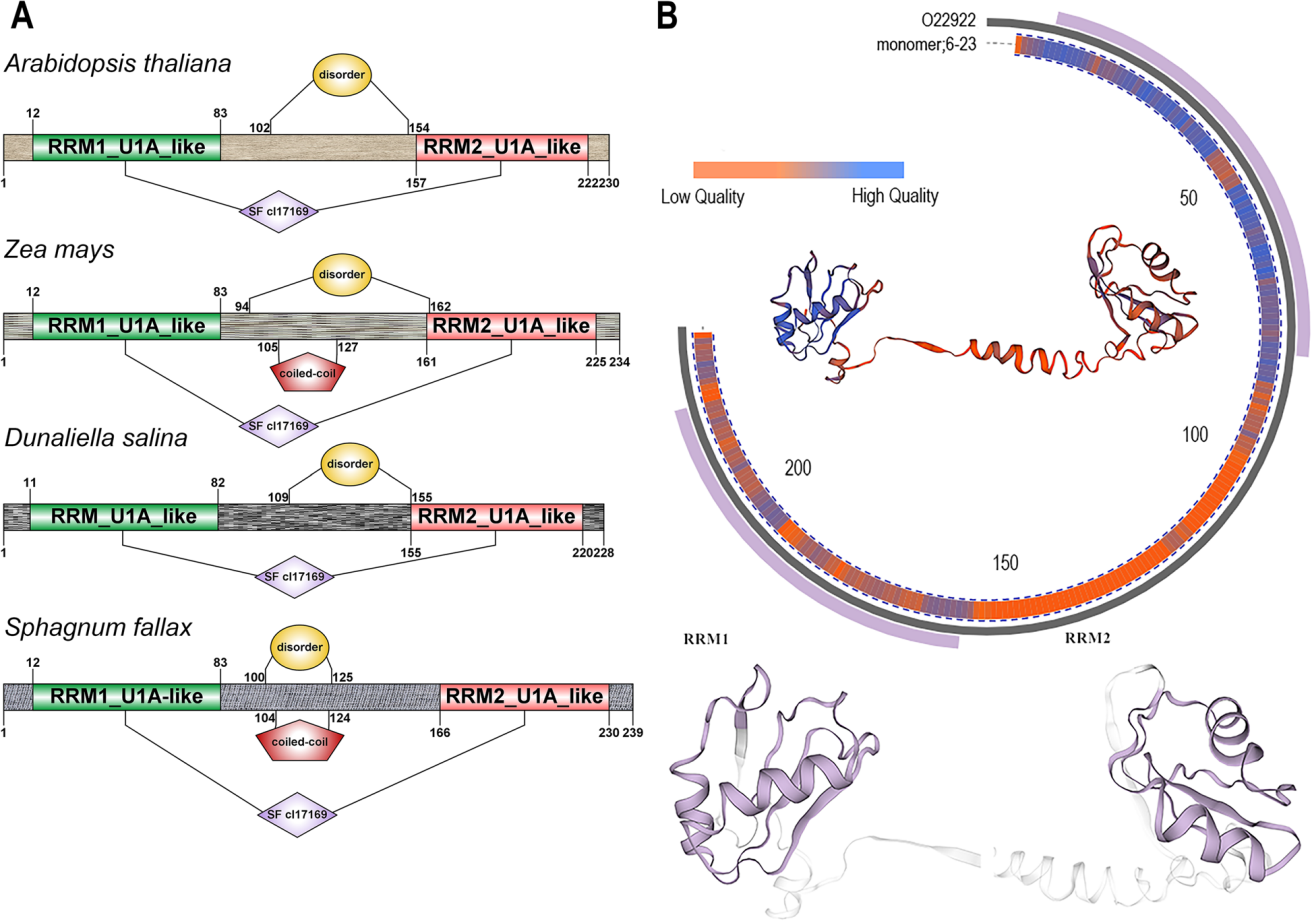
The conservative analysis of plant U2B″ proteins. (**A**) The structures of U2B'' proteins from different plants are displayed in different forms. (**B**) The 3D structure of plant U2B″ protein was generated (using *Arabidopsis thaliana* protein sequence) and represented. The outer ring represents RRM1 and RRM2 domains from top to bottom, the middle ring represents the template, and the inner ring represents U2B″ protein. The quality of the model is illustrated using a heat map in the inner ring

Since the protein domains were highly conserved, we estimated amino acid conservation. First, amino acid multiple sequence alignment was performed (Fig. S5). As expected, there was a high degree of conservation. The yellow, green, and white subfamilies differed from the other two subfamilies. The number of red amino acids was obviously greater, and the degree of red was greater, indicating that the yellow, green, and white subfamilies were closer together, whereas the pink and blue subfamilies were closer to one another, consistent with the results of the phylogenetic analysis. However, the two RRM domains of U2B″ also showed differences. The amino acid sequences of the RRM1 domain were highly conserved, whereas those of RRM2 were not as highly conserved. This may be related to the functions of the proteins, even though they all recognise the RNA. In addition, to demonstrate this point in more detail, we selected some plants (*A. thaliana, Zea mays, Dunaliella salina, Sphagnum fallax*) and plotted a two-dimensional map of their U2B″ proteins, which also revealed a high degree of conservation (Fig. 3A). Generally, the RRM1 domain is located between the 12th and 83rd amino acids, while the amino acid range of the RRM2 domain varies slightly between the 154th and 230th amino acids. Both RRM domains belong to the cl17169 superfamily (SF) and all proteins have disordered structures between the two domains. Furthermore, two had a coiled-coil structure.

A 3D model of plant U2B″ was constructed based on this template (Fig. 3B). The results of the conservative estimation of the 3D model were consistent with those of the amino acid multiple sequence alignment. RRM1 showed high quality, whereas RRM2 showed low quality.

### Promoter analysis

To further study the potential expression profiles of plant U2B'', the 1500-bp sequence upstream of the genes was analysed using PlantCARE. From 117 sequences, 12,885 elements were identified, including 1162 blank elements and 1339 unnamed elements, accounting for 9% and 10% of the total, respectively. Subsequently, the remaining elements were screened, and 1731 elements, accounting for 13% of the total, were removed. Finally, 8653 elements, accounting for 68% of the initial total, were included in the analysis. First, we classified the elements into four categories according to their function (Fig. S6A, Table S2), hormone response (879 elements), light response (541 elements), regulation of basic transcription (5932 elements), and stress response (1186 elements). An additional element was not clearly defined (115 AAGAA-motif). Hormone-responsive elements included 252 ABA-responsive elements (ABREs), 177 as-1 motifs, 177 CGTCA motifs, 96 estrogen response elements (EREs), and 177 TGACG motifs. Notably, three of five motifs had the same number, although whether they corresponded to each other remains to be further studied. The light-responsive elements comprised 162 BOX4, 288 G-box, and 91 TCT motifs. The most abundant regulatory elements of basic transcription included three common elements:2848 TATA boxes, 2768 CAAT boxes, and 316 AT-TATA boxes. The remaining stress-responsive elements were divided into four categories:143, 523, 316, and 204.

**Fig. 4:**
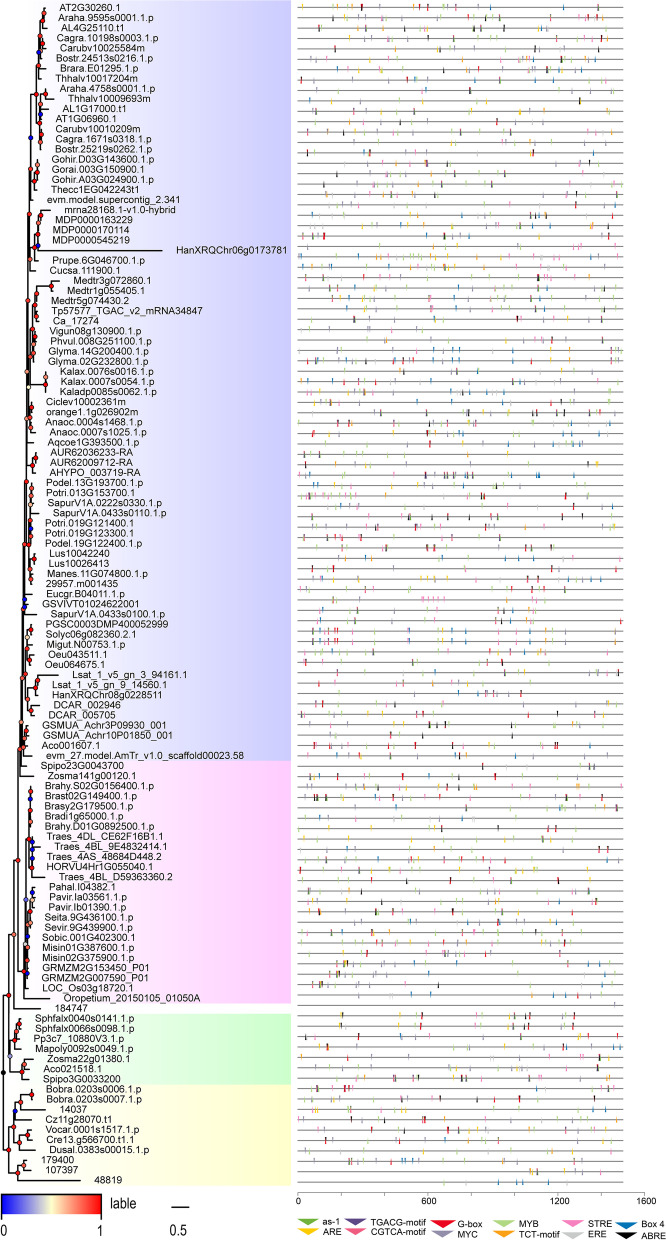
Summary of motifs that putatively occur in the promoter regions of plant *U2B″*. Triangles with various colours represent motifs. Motifs on the positive strand are labelled with an inverted triangle, whereas motifs on the negative strand with a normal triangle. The x-axis represents the length of the promoter sequence. Blue for dicotyledons, pink for monocotyledons, white for ferns, green for bryophytes, and yellow for algae

**Fig. 5:**
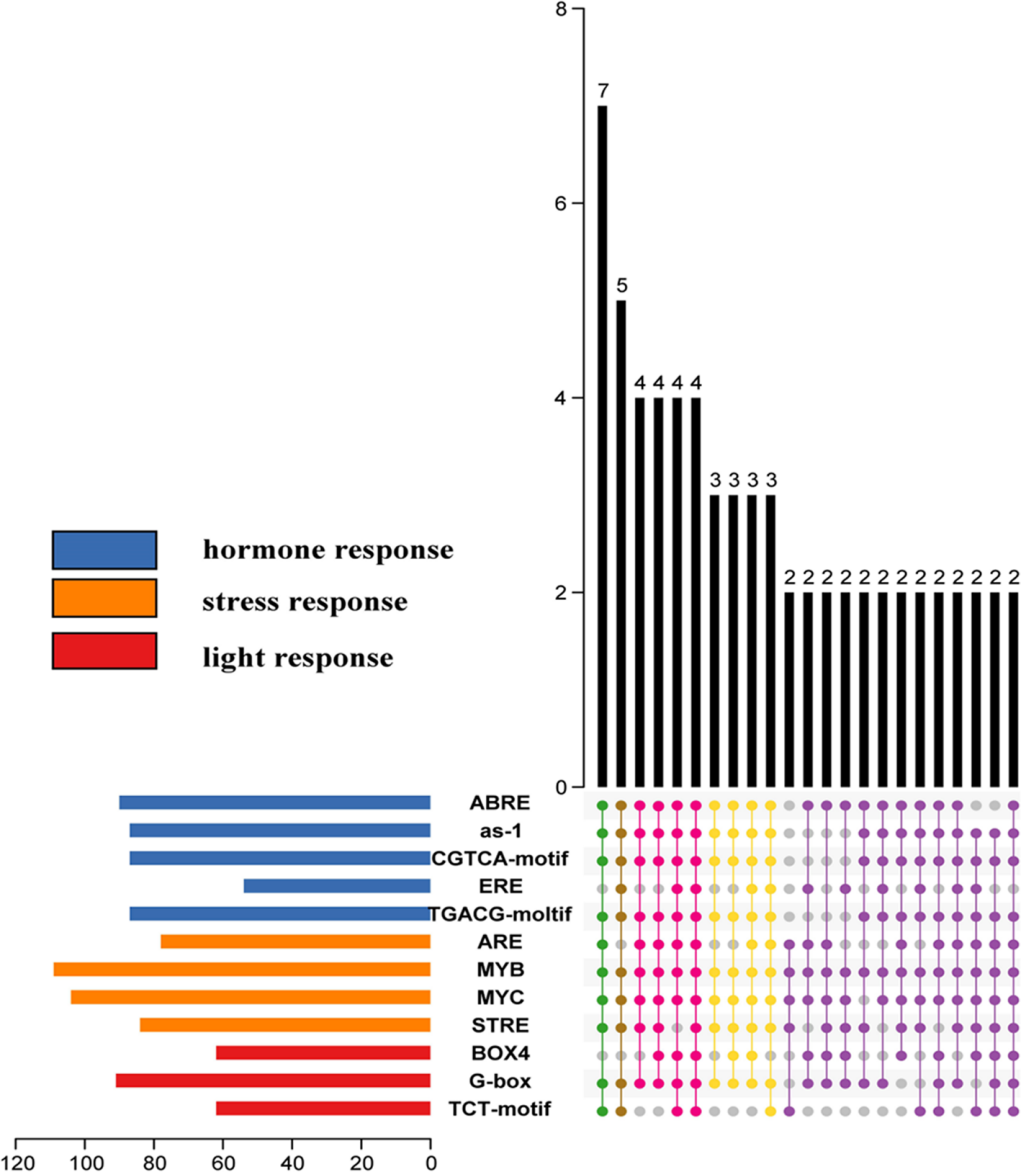
Specific enrichment statistics of the motifs in response to stress, hormones, and light. The point indicates that the species has this element. A point-to-point connection means having these elements at the same time. The number on the histogram shows the number of elements contained in the set. The x-axis represents the number of elements

We then combined the distribution map of elements (Fig. 4, right panel) on the sequence with the phylogenetic tree (Fig. 4, left panel). Excluding the white subfamily, the distribution of various elements in different subfamilies was relatively uniform. In general, the blue and pink subfamilies were distributed more than the other two subfamilies (Table S3). Owing to the considerable differences among family members, the number of promoters in the blue and pink subfamilies is also discrete. This may be due to differences in the degrees of evolution and development of different subfamilies. Notably, the numbers of elements in some sequences (*Oropetium thomaeum, Ostreococcus lucimarinus, Micromonas sp. RCC299*) were significantly lower than those in other sequences, indicating that they can only respond to a small number of stimuli. By contrast, they would have a single means of regulating gene expression. Subsequently, we performed an enrichment analysis of the stimuli of the sequence response (Fig. 5 and Fig. S6B). Among the 117 sequences, 112 responded to hormones, 113 to light, and 115 to stress, accounting for 96%, 97%, and 98%, respectively. Among them, 109 sequences (Fig. S6A) responded to the three stimuli simultaneously, exhibiting very high correlation. Notably, each group had two sequences that could respond to two of the three stimuli. Surprisingly, three sequences from three different plants (*Amaranthus hypochondriacus, Arabidopsis lyrata, Populus trichocarpa*) could only respond to stress. A specific analysis was then performed (Fig. 5). Surprisingly, only four sequences contained all elements at the same time. However, there were 49 sequences with 9–11 elements and 15 sequences with 5–8 elements simultaneously. Overall, the findings suggest that the *U2B″* can respond to diverse stimuli and has a complex response network.

### Subcellular localisation

**Fig. 6:**
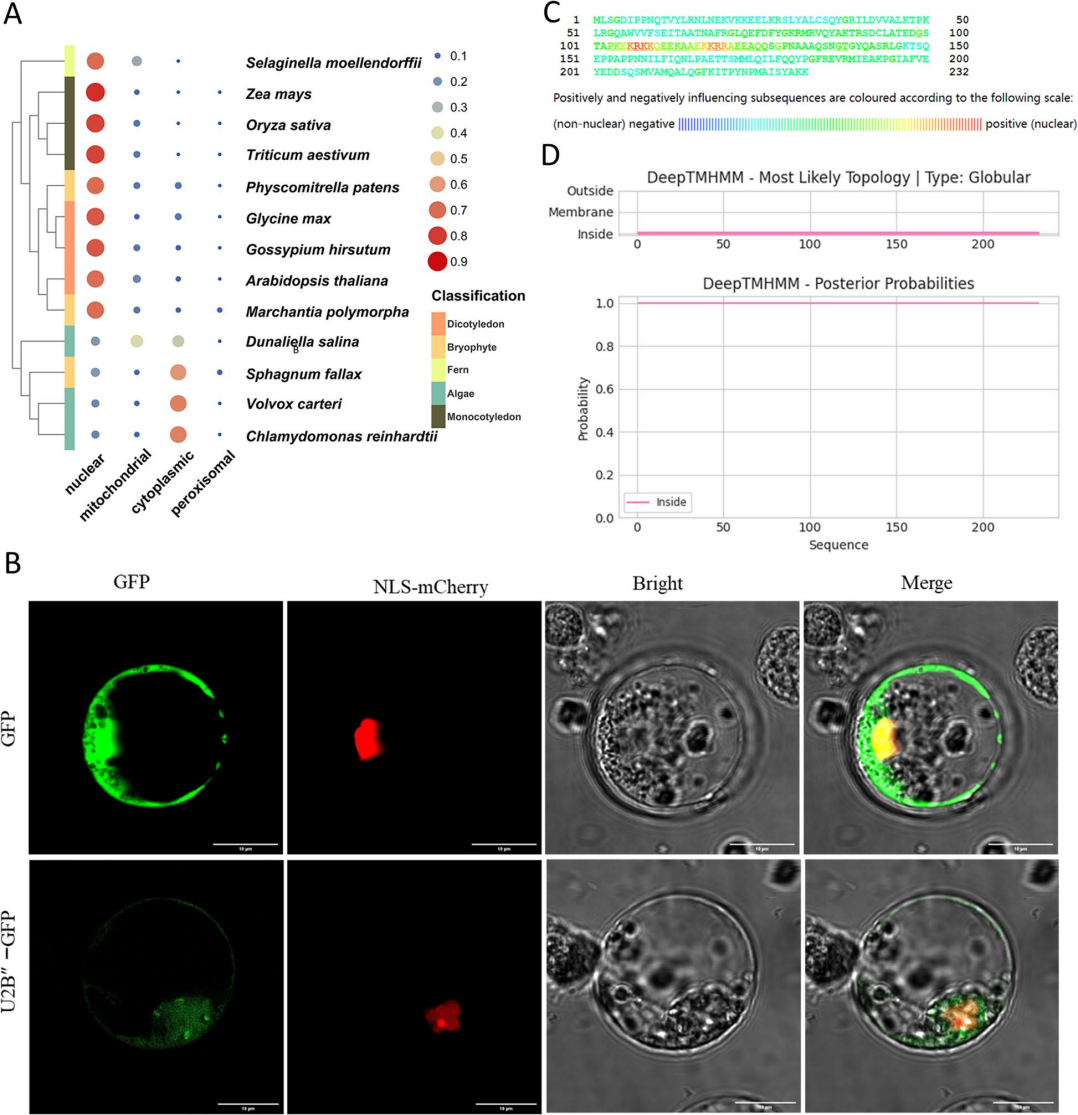
Subcellular localisation of U2B″-GFP*.* (**A**) Prediction results of subcellular localisation of U2B″ protein in different species. The larger the circle, the more likely it is to be positioned in that place. (**B**) Rice protoplast were selected as observation objects. U2B″-GFP and mCherry (nuclear localisation marker) were co-transferred into protoplast to observe the localisation of proteins. The white arrow marks the nucleoli. The experiment was repeated more than three times. (**C**) Nuclear localisation prediction of rice U2B″ protein. (**D**) Prediction of transmembrane structure of rice U2B″ protein

Knowledge of the subcellular localisation of proteins is also essential for the understanding of their functions. To further understand the function of U2B″, we performed subcellular localisation analysis to identify where U2B″ functions inside the cell (Fig. 6). First, we used web tools to predict the subcellular localisation of U2B″ in several species. The results were intriguing. In lower plants, U2B″ tends to be located in the cytoplasm, whereas in higher plants, U2B″ tends to be located in the nucleus (Fig. 6A). This means that over the course of evolution, the functions of U2B″ may have diverged based on its distinct positions. Considering that it is a component of the spliceosome and that splicing is essentially completed in the nucleus, we speculated that it may be located in the nucleus. To test this, we selected rice, which is one of the world’s most important crops. NLS-mCherry (a nuclear localisation marker) was selected as the control, to observe whether U2B″ was present in the nucleus. As expected, both were located in the nucleus. U2B″ and mCherry exhibited strong fluorescence, and a yellow signal was observed when the signals were merged (Fig. 6B). Notably, mCherry also had a strong localisation signal in the nucleolus, but U2B″ did not. This indicates that U2B″ plays a role in the nucleoplasm or nuclear membrane. Sequence analysis showed that the subsequences "KRKK" and "KKRR" in the junction structure between the two RRMs mainly promoted U2B″ to locate in the nucleus (Fig. 6C). The two RRMs are both disordered structures, suggesting that the structure could influence the positioning behaviour of U2B″. In addition, U2B″ has cytoplasmic localisation signals. Analysis of the transmembrane domain showed that U2B″ was within the cell, indicating that it had no localisation signal on the cell membrane (Fig. 6D).

### Expression of plant U2B″ genes

**Fig. 7:**
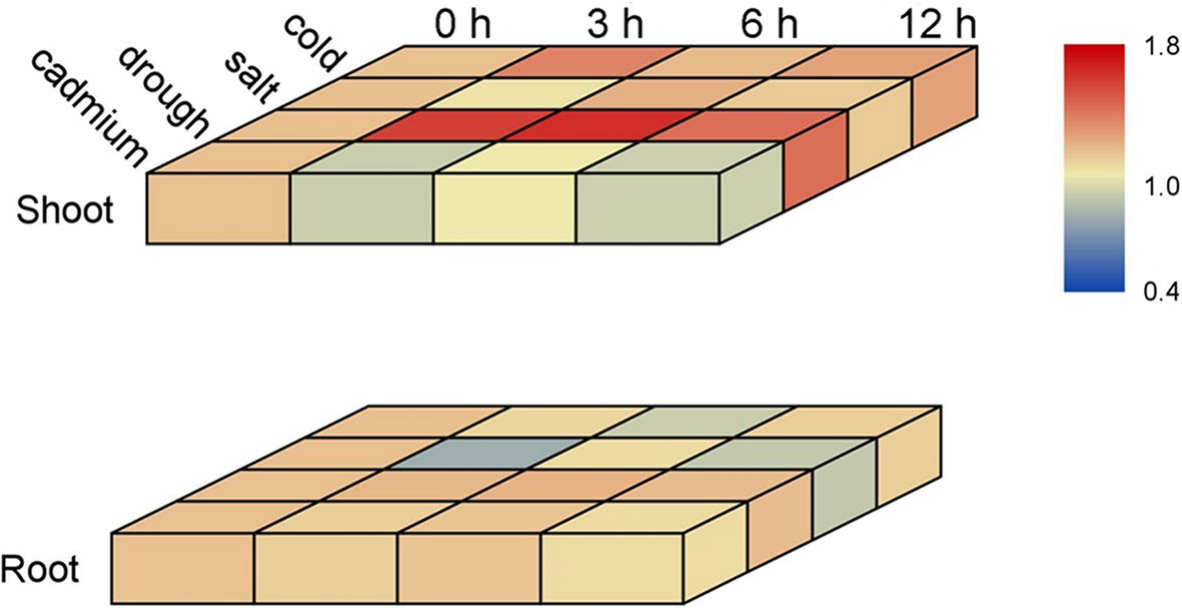
Expression of representative *U2B″* in *Oryza sativa*. *LOC_Os03g18720.1* was selected as *U2B″* in rice, and the relative expression data were transformed into a heatmap, which was divided into aboveground parts and underground parts. The data were log-transformed (base = 2) to generate a heatmap. All experiments were repeated at least three times

qPCR was performed to further study the levels of expression of *U2B″* in plants. Based on the results of promoter analysis, we selected four stress treatments: cold, drought, salt, and heavy metal cadmium (in the text referred to as Cd or Cd^2+^) (Fig. 7, Table S4). In general, excluding Cd stress, the levels of expression in the aboveground parts were higher than those in the belowground parts. The results suggest that U2B″ has contrasting regulatory mechanisms in the roots and shoots. Notably, there were not only differences in the levels of expression in the two parts but also contrasting expression patterns. U2B″ was not sensitive to Cd^2+^ and drought in roots, but showed significant differences in the shoot. U2B″ expression began to increase significantly after 3 h of drought treatment, which was sustained for 6 h. At 6 h, the relative expression was twice that of the control. However, it began to decline after 12 h of drought stress treatment. In contrast, the level of expression was significantly downregulated after 3 h of Cd treatment, and the relative expression was only 59% that of the control. However, it began to increase after 6 h and reached a level of significant difference, which was 77% of that of the control. After 12 h of Cd treatment, it began to decrease and finally reached the same level as 3 h, which was 60% of the control. When rice was treated with cold and salt, both roots and shoots exhibited sensitivity. Although they all exhibited sensitivity to stress, their response trends were distinct. Root and shoot exhibited similar trends under the salt treatment; both decreased first (40% and 81% of the control, respectively), increased (85% and 111% of the control, respectively), and then declined (56% and 95% of the control, respectively). However, the difference was that U2B″ was more responsive in the root. The difference between the salt treatment and the control was at most 60% in the root, whereas in the shoot, it was only 19%. In addition, U2B″ showed contrasting response patterns in the roots and shoots when subjected to cold stress. The levels of expressions were the lowest in the roots after 6 h of chilling treatment, and the highest in shoots after 3 h of chilling treatment.

### Alternative splicing profile analysis and splicing isoforms

Four representative monocotyledons, four dicotyledons, four algae, and three bryophytes were selected for this study. By comparing their gene structures, we observed that AS was not common in the U2B″ families (Fig. S7). AS occurred three times in the selected monocotyledons, twice in dicotyledons, and once in bryophytes; however, it did not occur in algae. In addition, more than two occurrences of AS were non-existent in the plants. Among the 15 selected plants, five plants had AS, including twice in *Arabidopsis*, while the other four had single occurrences. In *Z. mays* transcripts, only one of the five exons was preserved after AS. In the other four splicing isoforms, there was no significant difference between the gene structure and representative transcript forms, with some sequence differences observed in the UTRs. Overall, U2B″ may not undergo large splicing changes and may act uniformly on substrates with conserved RRM motifs across isoforms.

### Interaction network

**Fig. 8:**
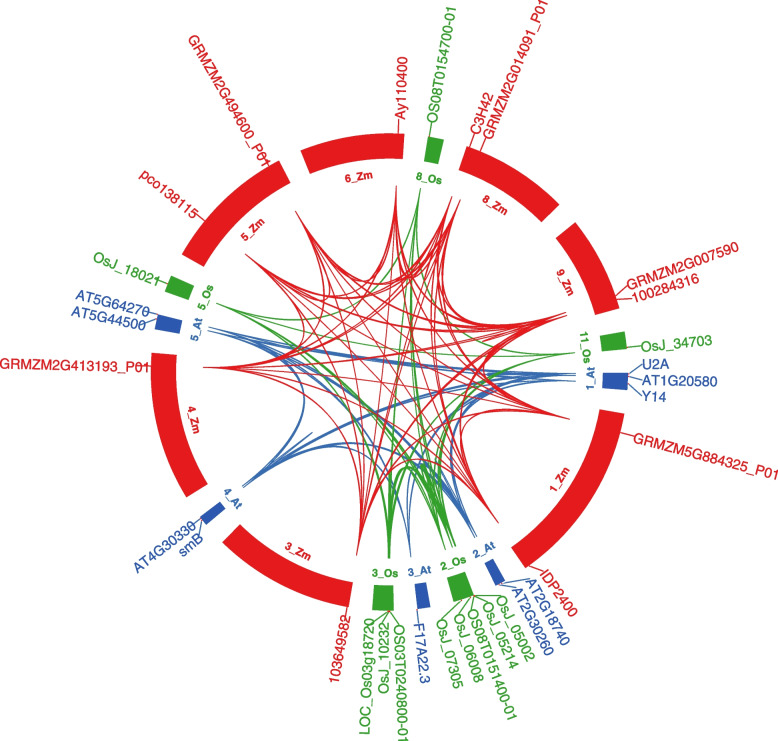
Protein interacting-partners of *Arabidopsis thaliana*, *Oryza sativa,* and *Zea mays* U2B″ proteins. Red represents *Z. mays*, green represents *O. sativa* and blue represents *A. thaliana.* The inner label represents the chromosomes, and the outer label represents the genes encoding the corresponding proteins. The line represents an interactive relationship

U2B″ needs to be translated into a protein to perform its function. We selected three common plants (*Arabidopis thaliana, Oryza sativa, Z. mays)* and constructed an interaction network (Fig. 8, Table S5) based on data obtained from STRING. We obtained 9, 10, and 10 interacting proteins in *A. thaliana*, *O. sativa*, and *Z. mays*. The numbers of interacting proteins showed clear consistency, suggesting that the U2B″ protein has a fairly complex interaction network in different plants. Regarding the genes encoding the corresponding proteins, there was an interesting phenomenon in which genes in *Z. mays* are evenly distributed on chromosomes, whereas those in *A. thaliana* and *O. sativa* were concentrated on a few chromosomes. Furthermore, among the nine proteins interacting with *AT2G30260*, there were nine snRNP proteins, one RNA-binding protein, and one putative splicing factor. Excluding a protein in *Z. mays* that has an unknown function, there were eight and seven snRNP proteins in *O. sativa* and *Z. mays*, respectively. In addition, both *O. Sativa* and *Z. mays* had an Sm-like protein, and it should be noted that the interaction protein in *Z. mays* also contains a zinc ion binding protein. In terms of the types of interacting proteins, the U2B″ protein showed a high degree of consistency in different plants. Notably, each plant has a unique interaction protein that differs from that of other plants. This may be caused by different degrees of evolution and development.

## Discussion

Structural analysis of the spliceosome has long been considered one of the most promising research fields in structural biology. Several human diseases can be attributed to incorrect splicing of genes or regulation of spliceosomes. In addition, 35% of genetic disorders in humans are caused by the variable splicing of single genes caused by gene mutations [37]. Some diseases are caused by mutations in splice proteins that affect the splicing of many transcripts. Some cancers are also involved in incorrect regulation of splicing factors [38, 39]. In a previous study, a small percentage of medulloblastoma samples had the same non-coding mutations. Following a preliminary study, the authors were surprised to find that this mutation (A > G) affected more than a small number of people, as nearly 100% of the adult patients with a certain disease subtype had the mutation. In addition, another mutation (A > C) in the same genome position is mainly present in liver cancer and chronic lymphoid leukaemia. Surprisingly, the mutation is located in the RNA-binding site of an important splicing RNA (U1 snRNA). Although at present, research on splicing mainly focuses on humans, there are still many reports related to plants. Some studies have found that the deletion or mutation of the mu1a gene of *Magnaporthe* (*M. oryzae* U1A, MU1A) may lead to abnormal splicing of the precursor mRNA, affecting the normal expression of some proteins, normal growth and metabolism processes, and ultimately, the pathogenicity of *Magnaporthe.* In the defence response to fungal infection, AS may be as important as traditional transcriptional control in *C. sublineola*-inoculated sorghum seedlings, as reported in a study based on next-generation sequencing technologies [40]. To screen for pathogenic effectors that regulate plant AS, a fluorescence reporting system was established to screen nine splicing regulation (SREs) effectors from 87 effectors of *Phytophthora infestans* [41]. Further studies have shown that SRE3 in combination with U1-70 K physically manipulates the plant AS mechanism, thereby regulating AS-mediated plant immunity. The biological clock of *Arabidopsis* not only controls gene transcription but also influences its post-transcriptional regulation by influencing AS [42]. Six differential AS events occurred in the defence response of germs in mock-inoculated and *S. sclerotiorum*-inoculated susceptible and tolerant *B. napus* plants, as determined by an analysis of 18 RNA-seq libraries [43]. However, the potential mechanism via which splice proteins affect splicing regulation remains unclear. The work described in this study provides an introductory layout for such works in future.

In the present study, we identified 117 U2B″ genes in 80 plant species. In the phylogenetic tree, plants with higher degrees of evolution and development were closely clustered together, and plants with lower degrees of evolution and development were similarly closely clustered together. Surprisingly, U2B″ in different plants showed consistently high conservation in multiple analyses. Nearly 70% of the sequences showed a 5 exon-2 UTR structure, and 90% of the sequences were predicted to contain an N-terminal domain named RRM1 and a C-terminal domain annotated as RRM2. Generally, if a gene is highly conserved in many species, it is believed that these similarities across species indicate that the gene performs some basic functions necessary for many life forms, and therefore retains these sequences during evolution. These sequences are generally necessary for life activities, and mutations in these sequences often lead to death. Therefore, few mutations have been preserved during evolution, so that the genes are highly conserved. The same is true for U2B″, which may perform one or more functions essential for life. We prefer U2B″ to perform a single or a few functions essential for life. *U2B''* knockout did not lead to a lethal phenotype in worms [32]. U1A replaces snRNP and binds to its corresponding snRNP. However, we found that the N- and C-terminal domains of the protein were different in the multisequence alignment. Although both domains can recognise RNA, the N-terminus is more conserved than the C-terminus. We speculate that RRM1 performs the most basic functions required by all species, whereas RRM2 performs distinct functions in evolution and development.

The U2 snRNP is a subcomplex in early spliceosomal assembly. U2B″ plays an important role in the snRNP complex. However, very few studies have elucidated its biological function in plants. Promoter analysis revealed that according to the complex response elements, U2B″ could respond to various stimuli. First, many stress-responsive elements were identified, including AREs, MYBs, and STREs. As a necessary cis-regulatory element for anaerobic induction, AREs play an important role in plants. MYB-MYC is a plant-specific element widely involved in plant growth, development, and response to abiotic stress. STRE is reportedly involved in a variety of stress responses in Neurospora, including heat, osmotic, and oxidative stress, and the molecular mechanism mediated by STRE may not be conserved [44]. In addition to many stress response components, there are a large number of hormone-responsive elements upstream of U2B^″^, including ABRE, activating sequence-1, CGTCA motif, ERE, and the TGACG motif. ABREs are response elements of ABA [45] that play a variety of important roles in plants. In higher plants, AS-1 of the cauliflower mosaic virus 35S promoter mediates SA- and IAA-inducible transcriptional activation [46]. As MeJA-responsive elements, the CGTCA and TGACG motifs are found in a large number of plants. The estrogen response element (ERE) is a conserved DNA sequence in the promoters of the estrogen target genes. It can bind to estrogen receptors, is transcriptionally regulated, and is usually located in the promoters of its target genes [47]. These results indicate that U2B″ is involved extensively in plant growth, development, and stress responses. We also confirmed this using qPCR. However, the interaction protein of U2B″ is relatively simple. These include the major snRNPs common to the three plants and other minor proteins specific to each plant. This may be caused by different degrees of evolution and development. Overall, the above studies show that U2B″ can respond to a variety of external stimuli but can only interact with a limited number of proteins.

## Conclusion

In the present study, 117 U2B″ genes were identified in 80 plant types. Comprehensive bioinformatic analysis and partial experiments of the gene family were performed. Considering that U2B″ is highly conserved in different plants and is located in the nucleus, it can respond to a variety of external stimuli, indicating that it is extensively involved in plant growth and development. However, the mechanism by which U2B″ regulates plant growth and development requires further study.

## Methods

### Identification of plant U2B″

The *A. thaliana* U2B″ protein (*AT2G30260*) was used as the original sequence for carrying out a protein BLAST search with an e-value cutoff of 1e-10 [48] against 80 plant genome sequences from Phytozome v12.1. Consequently, 117 putative U2B″/U2B″-like sequences were identified for subsequent research.

### Phylogenetic analysis

Protein sequences of the plant U2B″ identified above were obtained for phylogenetic tree construction to infer clustering patterns and evolutionary relationships. The transcript with the longest coding sequence was selected for loci with multiple splicing subtypes. Subsequently, multiple sequence alignment of proteins were performed using Muscle V3.8 with the default settings [49]. A phylogenetic tree was constructed using MrBayes v3.2.2 (Jones model) [50].

### Analysis of gene and protein structure

The structural information of genes and proteins was obtained from the Phytozome v12.1 and Pfam protein family databases. The top 10 detected motifs were obtained using default settings on the MEME server from the cDNA and protein sequences of plant U2B″ [51].

### Promoter motif prediction and the protein–protein interaction network

The 1.5-kb promoter sequences of plant U2B″ were selected from the Phytozome database and used for the prediction of cis-elements using PlantCARE [52]. *Arabidopsis* U2B″ protein was input to STRING and PlantSPEAD [53] to find the most interacting partners.

### Subcellular localisation

The CDS of *OsU2B″* (*LOC_Os03g18720.1*) was amplified using gene-specific primers (5'-CCTGTTGTTTGGTGTTACTTAAGCTTATGTTGTCCGGCGACATACC-3' and 5'- TCCTCGCCCTTGCTCACCATGGATCCTCACTTCTTTGCGTAGGATATAGCC-3') and inserted into the GFP (pGreen II-UBI-GFP) vector to generate a fusion construct according to Lu et al. [1]. NLS − mCherry (SV40 large − T antigen NLS, a signal peptide guiding GFP into the nucleus) was selected as a marker for nuclear localisation [54]. Nipponbare rice seedlings grown at approximately 30 °C for one week were selected, and their stems were collected to extract protoplasts. Equal volumes of the constructed plasmids and extracted protoplasts were mixed with 40% PEG4000 solution at a ratio of 1:5 (v/v), cultured overnight at 30 °C, and observed under a laser confocal microscope (Leica TCS SP8) more than three times. The subcellular localisation of U2B″ in some species was predicted using the online PSORT database. NucPred was used to predict the nuclear localisation of protein sequences [55]. DeepTMHMM was used to predict the protein transmembrane domains.

### Plant growth and RT-qPCR analysis

Nipponbare seeds were first soaked in carbendazim for one day and then in water for another day. The seeds were then transferred to the climate chamber and subjected to stress when they grew two true leaves (22 d). Materials were grown at 22 °C/20 °C under a 16 h/ 8 h light/dark photoperiod and a light intensity of 120 μmol m ^−2^ s ^−1^ in an incubator (QY-14; Nanjing Quanyou Electronic Technology Co., Ltd., China). Hydroponics was used in all four stress treatments, including cold treatment at 8 °C and drought treatment, with 20% PEG6000, salt treatment with 100 mmol L^−1^ sodium chloride, and heavy metal cadmium treatment with 100 μmol L^−1^ cadmium sulfate. Total RNA was isolated using TRIzol reagent (Invitrogen) and converted to complementary DNA (cDNA) using PrimeScript™ RT Master Mix (TAKARA) according to the manufacturer’s protocol. RT-qPCR was performed using a 7500 Real-time PCR Detection System (Bio-Rad) in conjunction with SYBR Fast qPCR Mix (TAKARA) and was repeated at least three times. Forward and reverse primers of *OsU2B″* were used to produce a single amplification (5'- GCAACCGAAGATGGTTCTACTG -3' and 5'- GCTTTGGGCAGCAGCATTAG -3'). Gene expression was presented as a heat map using TBtools v1.048 [56]. *OsACTIN* (*LOC_Os05g36290*) was used as a reference gene.

### Model construction and amino acid conservation assessment

Homology modelling was carried out using the *A. thaliana* U2B″ protein sequence (UniProtKB AC: O22922) based on the template 4pkd.1. B using SWISS-MODEL [57, 58]. Amino acid multiple sequence alignment and conservation scores based on frequency-based differences were obtained using the default values from NCBI.

## Supplementary Information

**Additional file 1:** **Fig. S1.** Formation of mature 17S U2 snRNP. The upper one is12S core particle, the middle one is 15S pre-mature particle, and the lower oneis 17S functional maturity particle. Continuous thin black lines represent U2 snRNA. **Fig. S2.** The design process of the whole article. **Fig. S3.** Circle phylogenetic tree representation of the available plant U2B″ gene family. Phylogenetic analysisof plant U2B″ gene family was carried out by using software MrBayes v3.2.2. The posterior probability values are labeled at each major branch. Blue for dicotyledons, pink for monocotyledons,white for ferns, green for bryophytes, and yellow for algae. **Fig. S4.** Motifs of genomic structure and protein structure analysis. (**A**)  Consensus sequence of top ten identified DNA motifs are listed in ascending order. (**B**)  Consensus sequence of top ten identified amino-acid motifs are listed in ascending order. **Fig. S5.** The multiple sequence alignment of RRM domains for the conservative analysis. The sequences are arranged from top to bottom in phylogenetic tree. **Fig. S6.** Promoter classification and enrichment analysis. (**A**) Statistics of motifs function and number. The x-axis represents the number of elements. (**B**)Overall enrichment statistics of motifs in response to stress, hormones and light.**Fig. S7.** AS profile analysis. Summary of annotated alternatively spliced transcript isoforms for identified U2B″ genes. Pink represents monocotyledons, blue represents dicotyledons, green represents bryophytes, and yellow represents algae.

**Additional file 2:** **Table S1.** Sequence summary of plant *U2B″* gene phylogenetic analysis.

**Additional file 3:** **Table S2.** Specific data of enrichment analysis.

**Additional file 4:** **Table S3.** Statistical analysis of promoter distribution.

**Additional file 5:** **Table S4.** Expression of *U2B″* in rice under different stresses. 

**Additional file 6:** **Table S5.** Sequence summary of plant U2B″ protein-protein interaction network.

## Data Availability

All the data generated during this study are included in this published article and its supplementary files. The data used and/or analyzed during the current study are available from the Phytozome (https://phytozome-next.jgi.doe.gov/pz/), PlantCARE (http://bioinformatics.psb.ugent.be/webtools/plantcare/html/), STRING (https://string-db.org/), PlantSPEAD (http://chemyang.ccnu.edu.cn/ccb/database/PlantSPEAD/index.php/home/index), PSORT (https://www.genscript.com/psort.html), DeepTMHMM (https://dtu.biolib.com/DeepTMHMM), UniProtKB (https://www.uniprot.org/), SWISS-MODEL (https://swissmodel.expasy.org/) and NCBI (https://www.ncbi.nlm.nih.gov/) website.

## Abbreviations

AS: Alternative splicing
ABREs: ABA-responsive elements
ERE: Estrogen response element
ESE: Exon splicing enhancer
ISE: Intron splicing enhancer
LSm: Like-Sm
RRMs: RNA recognition motifs
SR: Splicing regulatory
SREs: Splicing regulatory elements
SF: Superfamily
U2B”: U2 small nuclear ribonucleoprotein B
