# Supplementary material for: Phylogenetic analysis and stress response of the plant U2 small nuclear ribonucleoprotein B″ gene family
Source: BMC Genomics. 2022 Nov 8;23:744. doi: 10.1186/s12864-022-08956-0 (PMC9644473; doi:10.1186/s12864-022-08956-0)
Supplement: Supplementary file 1 — Additional file 1: Fig. S1. Formation of mature 17S U2 snRNP. The upper one is12S core particle, the middle one is 15S pre-mature particle, and the lower oneis 17S functional maturity particle. Continuous thin black lines represent U2 snRNA. Fig. S2. The design process of the whole article. Fig. S3. Circle phylogenetic tree representation of the available plant U2B″ gene family. Phylogenetic analysisof plant U2B″ gene family was carried out by using software MrBayes v3.2.2. The posterior probability values are labeled at each major branch. Blue for dicotyledons, pink for monocotyledons,white for ferns, green for bryophytes, and yellow for algae. Fig. S4. Motifs of genomic structure and protein structure analysis. (A) Consensus sequence of top ten identified DNA motifs are listed in ascending order. (B) Consensus sequence of top ten identified amino-acid motifs are listed in ascending order. Fig. S5. The multiple sequence alignment of RRM domains for the conservative analysis. The sequences are arranged from top to bottom in phylogenetic tree. Fig. S6. Promoter classification and enrichment analysis. (A) Statistics of motifs function and number. The x-axis represents the number of elements. (B)Overall enrichment statistics of motifs in response to stress, hormones and light.Fig. S7. AS profile analysis. Summary of annotated alternatively spliced transcript isoforms for identified U2B″ genes. Pink represents monocotyledons, blue represents dicotyledons, green represents bryophytes, and yellow represents algae. [file 12864_2022_8956_MOESM1_ESM.docx]

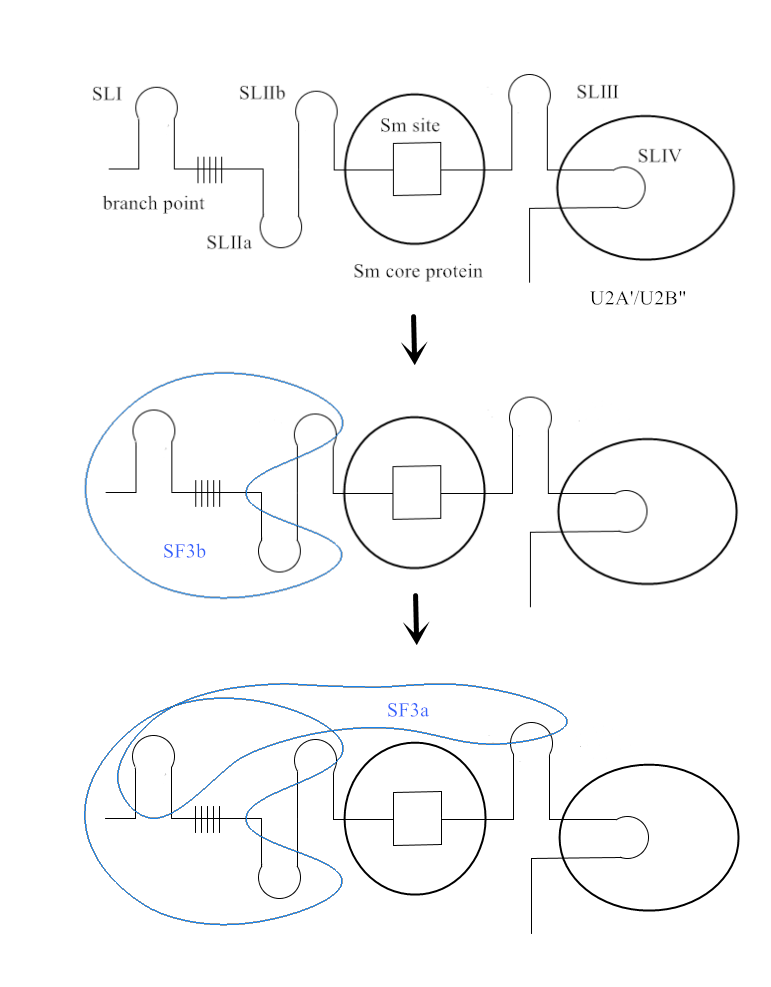


**Fig. S1** Formation of mature 17S U2 snRNP. The upper one is 12S core particle, the middle one is 15S pre-mature particle, and the lower one is 17S functional maturity particle. Continuous thin black lines represent U2 snRNA.


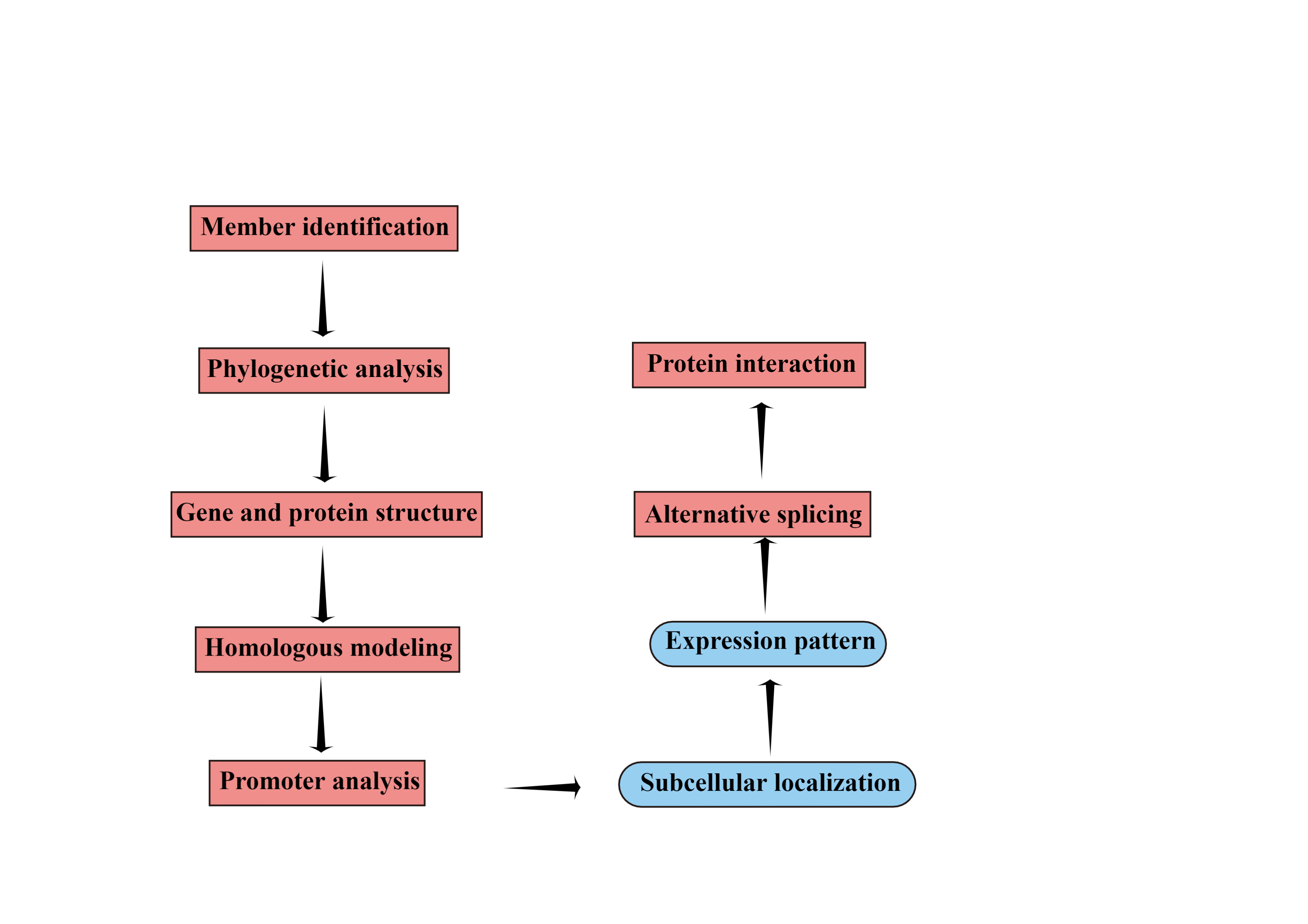


**Fig. S2** The design process of the whole article.


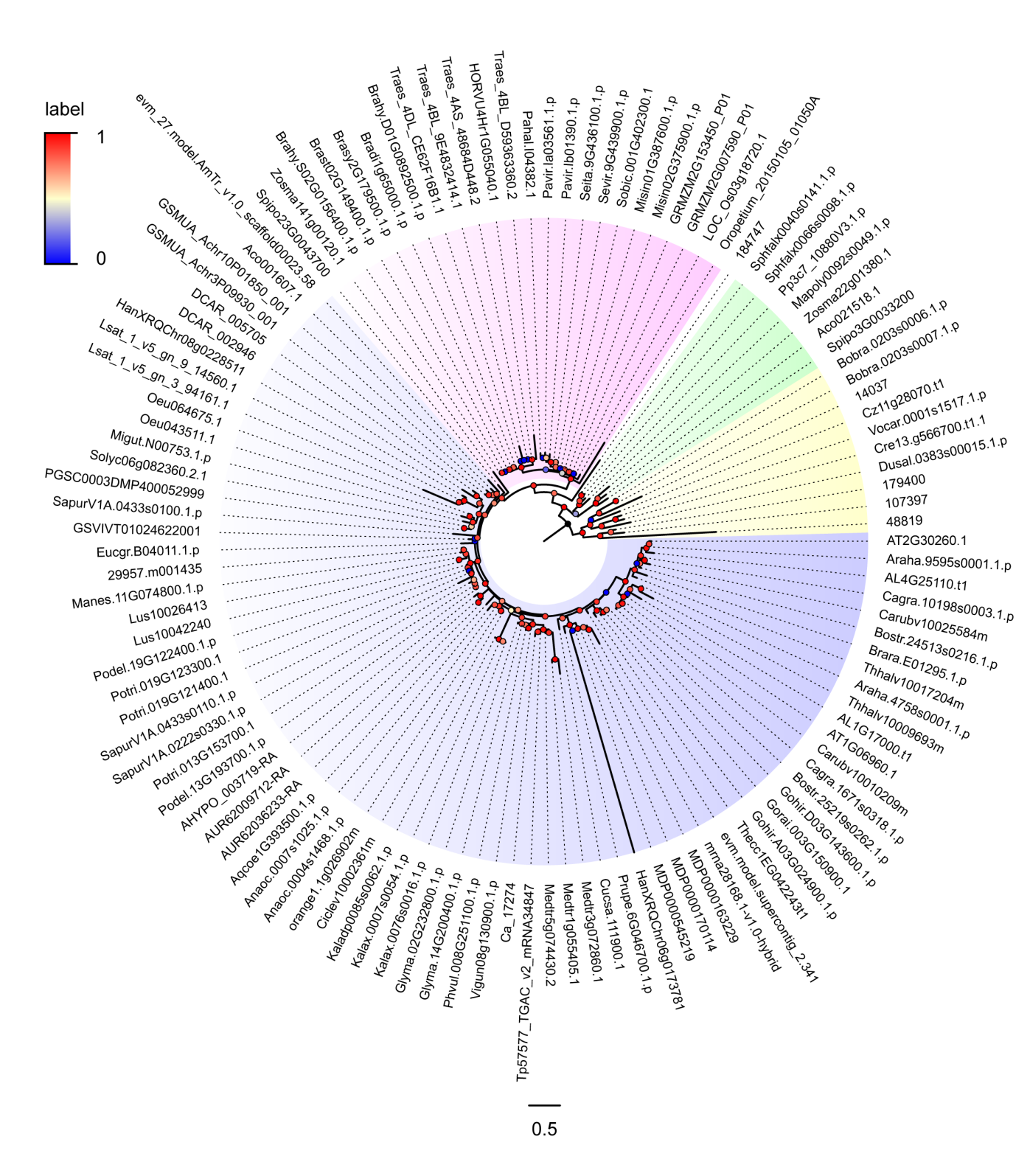


**Fig. S3** Circle phylogenetic tree representation of the available plant U2B” gene family. Phylogenetic analysis of plant U2B” gene family was carried out by using software MrBayes v3.2.2. The posterior probability values are labeled at each major branch. Blue for dicotyledons, pink for monocotyledons, white for ferns, green for bryophytes, and yellow for algae.

**
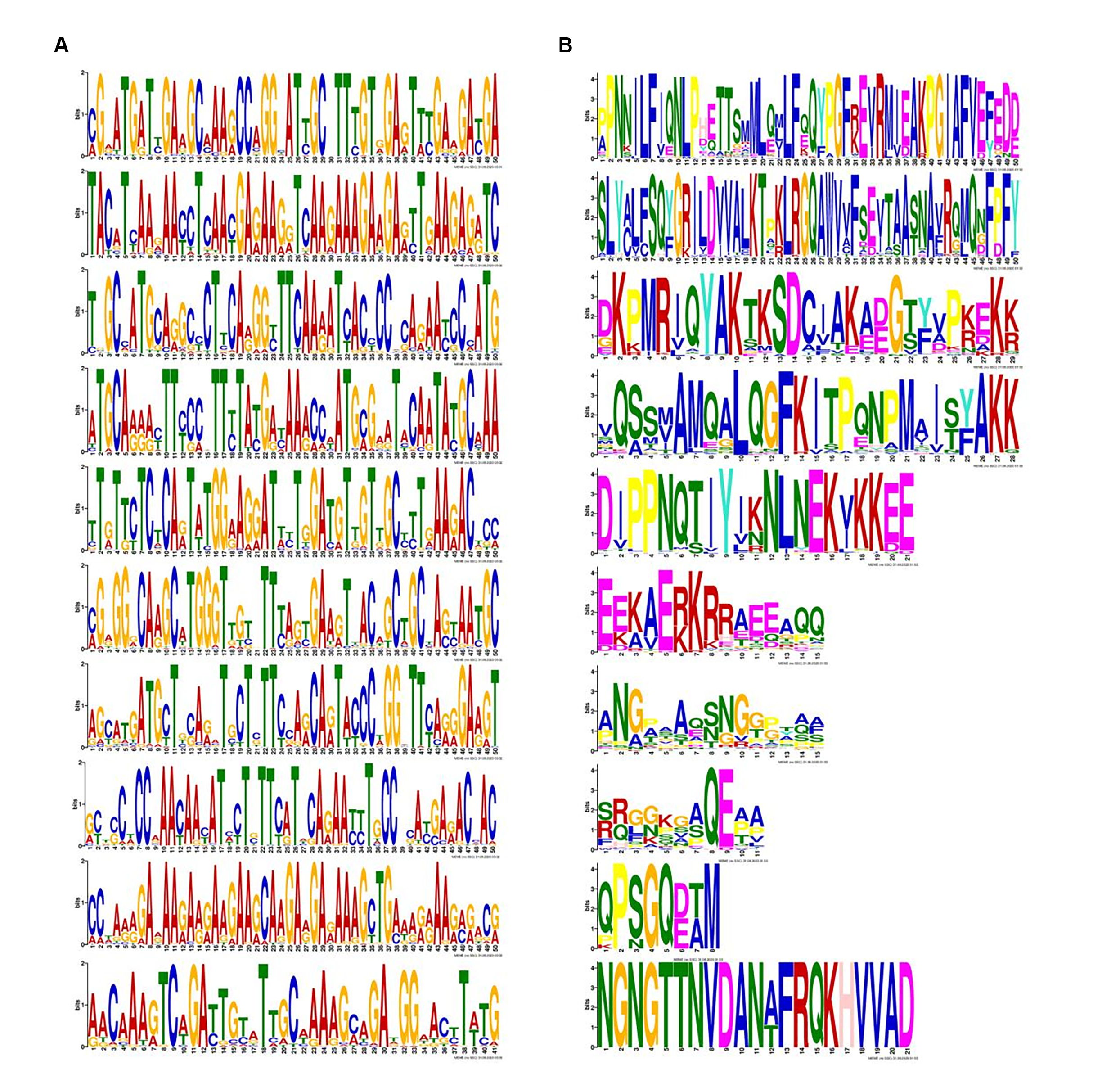
**

**Fig. S4** Motifs of genomic structure and protein structure analysis. (**A**) Consensus sequence of top ten identified DNA motifs are listed in ascending order. (**B**) Consensus sequence of top ten identified amino-acid motifs are listed in ascending order.

**

**

**Fig. S5** The multiple sequence alignment of RRM domains for the conservative analysis. The sequences are arranged from top to bottom in phylogenetic tree.

**
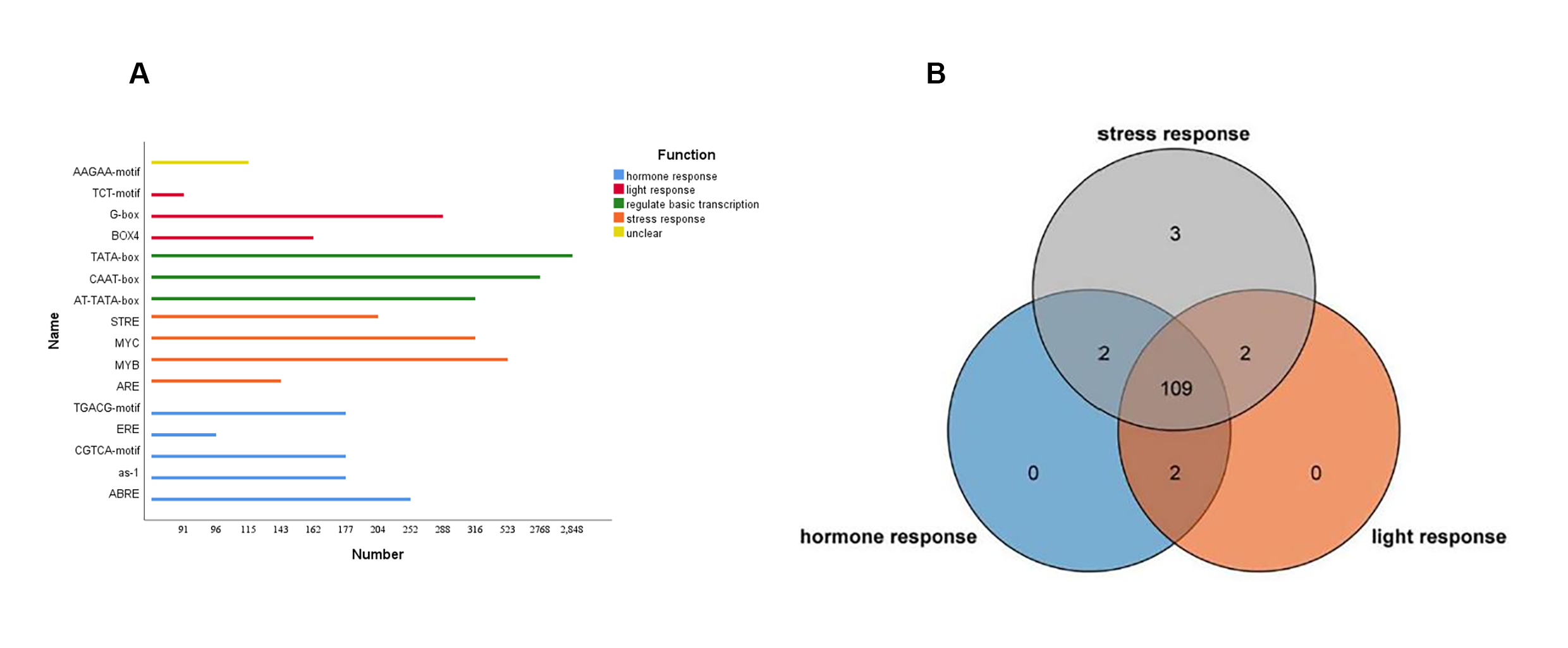
**

**Fig. S6** Promoter classification and enrichment analysis. (**A**) Statistics of motifs function and number. The x-axis represents the number of elements. (**B**) Overall enrichment statistics of motifs in response to stress, hormones and light.


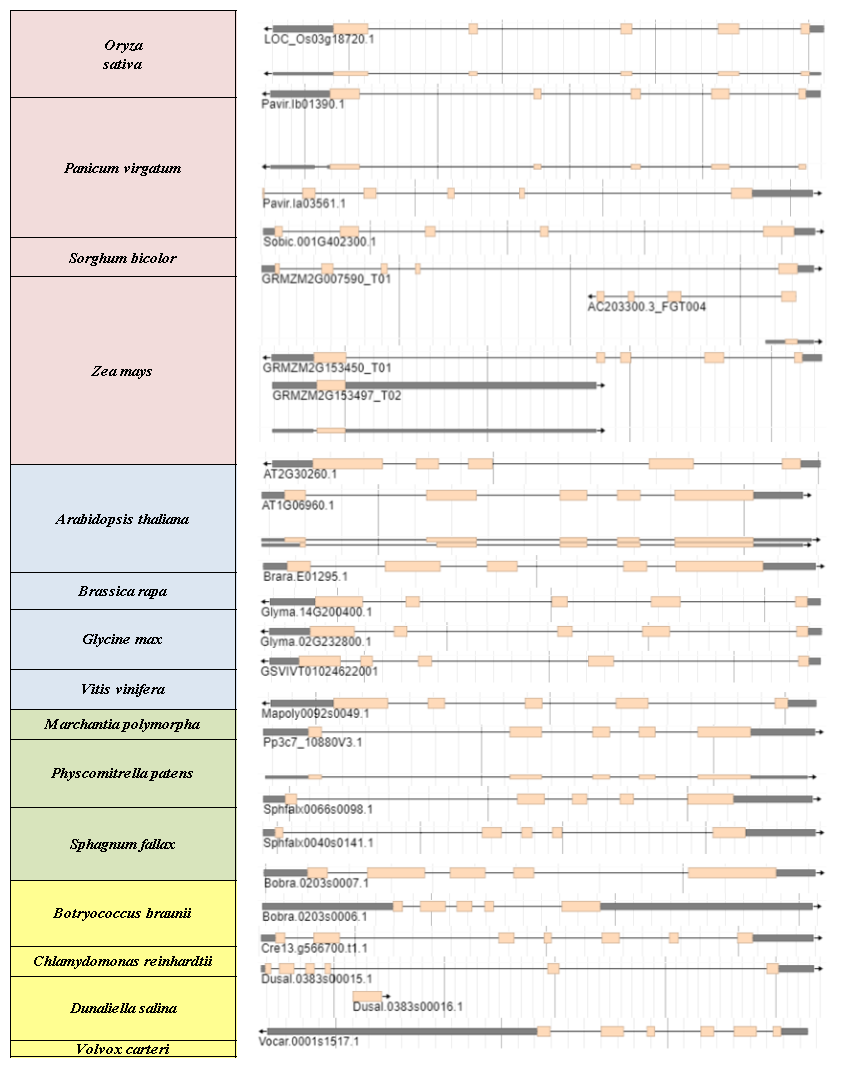
**Fig. S7** AS profile analysis. Summary of annotated alternatively spliced transcript isoforms for identified U2B” genes. Pink represents monocotyledons, blue represents dicotyledons, green represents bryophytes, and yellow represents algae.
