# Supplementary material for: Phylogenetic analysis and stress response of the plant U2 small nuclear ribonucleoprotein B″ gene family
Source: BMC Genomics. 2022 Nov 8;23:744. doi: 10.1186/s12864-022-08956-0 (PMC9644473; doi:10.1186/s12864-022-08956-0)
Supplement: Supplementary file 2 — Additional file 2: Table S1. Sequence summary of plant U2B″ gene phylogenetic analysis. [file 12864_2022_8956_MOESM2_ESM.docx]

**Table S1 Sequence summary of plant *U2B”* gene phylogenetic analysis.**

| Subfamily | Groups | Organism | Transcript ID | Gene ID |
| --- | --- | --- | --- | --- |
| Blue | Dicotyledon | *Amaranthus hypochondriacus* | AHYPO_003719-RA | AHYPO003719 |
|  |  | *Amborella trichopoda* | evm_27.model.AmTr_v1.0_scaffold00023.58 | evm_27.TU.AmTr_v1.0_scaffold00023.58 |
|  |  | *Anacardium occidentale* | Anaoc.0004s1468.1 | Anaoc.0004s1468 |
|  |  |  | Anaoc.0007s1025.1 | Anaoc.0007s1025 |
|  |  | *Aquilegia coerulea* | Aqcoe1G393500.1 | Aqcoe1G393500 |
|  |  | *Arabidopsis halleri* | Araha.4758s0001.1 | Araha.4758s0001 |
|  |  |  | Araha.9595s0001.1 | Araha.9595s0001 |
|  |  | *Arabidopsis lyrata* | AL1G17000.t1 | AL1G17000 |
|  |  |  | AL4G25110.t1 | AL4G25110 |
|  |  | *Arabidopsis thaliana* | AT1G06960.1 | AT1G06960 |
|  |  |  | AT2G30260.1 | AT2G30260 |
|  |  | *Boechera stricta* | Bostr.24513s0216.1 | Bostr.24513s0216 |
|  |  |  | Bostr.25219s0262.1 | Bostr.25219s0262 |
|  |  | *Brassica rapa* | Brara.E01295.1 | Brara.E01295 |
|  |  | *Capsella grandiflora* | Cagra.10198s0003.1 | Cagra.10198s0003 |
|  |  |  | Cagra.1671s0318.1 | Cagra.1671s0318 |
|  |  | *Capsella rubella* | Carubv10010209m | Carubv10010209 |
|  |  |  | Carubv10025584m | Carubv10025584 |
|  |  | *Carica papaya* | evm.model.supercontig_2.341 | evm.TU.supercontig_2.341 |
|  |  | *Chenopodium quinoa* | AUR62009712-RA | AUR62009712 |
|  |  |  | AUR62036233-RA | AUR62036233 |
|  |  | *Cicer arietinum* | Ca_17274 | Ca_17274 |
|  |  | *Citrus clementina* | Ciclev10002361m | Ciclev10002361 |
|  |  | *Citrus sinensis* | orange1.1g026902m | orange1.1g026902 |
|  |  | *Cucumis sativus* | Cucsa.111900.1 | Cucsa.111900 |
|  |  | *Daucus carota* | DCAR_002946 | DCAR_002946 |
|  |  |  | DCAR_005705 | DCAR_005705 |
|  |  | *Eucalyptus grandis* | Eucgr.B04011.1 | Eucgr.B04011 |
|  |  | *Eutrema salsugineum* | Thhalv10009693m | Thhalv10009693 |
|  |  |  | Thhalv10017204m | Thhalv10017204 |
|  |  | *Fragaria vesca* | mrna28168.1-v1.0-hybrid | mrna28168.1-v1.0-hybrid |
|  |  | *Glycine max* | Glyma.02G232800.1 | Glyma.02G232800 |
|  |  |  | Glyma.14G200400.1 | Glyma.14G200400 |
|  |  | *Gossypium hirsutum* | Gohir.A03G024900.1 | Gohir.A03G024900 |
|  |  |  | Gohir.D03G143600.1 | Gohir.D03G143600 |
|  |  |  | Gorai.003G150900.1 | Gorai.003G150900 |
|  |  | *Helianthus annuus* | HanXRQChr06g0173781 | HanXRQChr06g0173781 |
|  |  |  | HanXRQChr08g0228511 | HanXRQChr08g0228511 |
|  |  | *Kalanchoe fedtschenkoi* | Kaladp0085s0062.1 | Kaladp0085s0062 |
|  |  | *Kalanchoe laxiflora* | Kalax.0007s0054.1 | Kalax.0007s0054 |
|  |  |  | Kalax.0076s0016.1 | Kalax.0076s0016 |
|  |  | *Lactuca sativa* | Lsat_1_v5_gn_3_94161.1 | Lsat_1_v5_gn_3_94161 |
|  |  |  | Lsat_1_v5_gn_9_14560.1 | Lsat_1_v5_gn_9_14560 |
|  |  | *Linum usitatissimum* | Lus10026413 | Lus10026413 |
|  |  |  | Lus10042240 | Lus10042240 |
|  |  | *Malus domestica* | MDP0000163229 | MDP0000163229 |
|  |  |  | MDP0000170114 | MDP0000170114 |
|  |  |  | MDP0000545219 | MDP0000545219 |
|  |  | *Manihot esculenta* | Manes.11G074800.1 | Manes.11G074800 |
|  |  | *Medicago truncatula* | Medtr1g055405.1 | Medtr1g055405 |
|  |  |  | Medtr3g072860.1 | Medtr3g072860 |
|  |  |  | Medtr5g074430.2 | Medtr5g074430 |
|  |  | *Mimulus guttatus* | Migut.N00753.1 | Migut.N00753 |
|  |  | *Olea europaea var. sylvestris* | Oeu043511.1 | Oeu043511 |
|  |  |  | Oeu064675.1 | Oeu064675 |
|  |  | *Phaseolus vulgaris* | Phvul.008G251100.1 | Phvul.008G251100 |
|  |  | *Populus deltoides* | Podel.13G193700.1 | Podel.13G193700 |
|  |  |  | Podel.19G122400.1 | Podel.19G122400 |
|  |  | *Populus trichocarpa* | Potri.013G153700.1 | Potri.013G153700 |
|  |  |  | Potri.019G121400.1 | Potri.019G121400 |
|  |  |  | Potri.019G123300.1 | Potri.019G123300 |
|  |  | *Prunus persica* | Prupe.6G046700.1 | Prupe.6G046700 |
|  |  | *Ricinus communis* | 29957.m001435 | 29957.m001435 |
|  |  | *Salix purpurea* | SapurV1A.0222s0330.1 | SapurV1A.0222s0330 |
|  |  |  | SapurV1A.0433s0100.1 | SapurV1A.0433s0100 |
|  |  |  | SapurV1A.0433s0110.1 | SapurV1A.0433s0110 |
|  |  | *Solanum lycopersicum* | Solyc06g082360.2.1 | Solyc06g082360.2 |
|  |  | *Solanum tuberosum* | PGSC0003DMP400052999 | PGSC0003DMP400052999 |
|  |  | *Theobroma cacao* | Thecc1EG042243t1 | Thecc1EG042243t1 |
|  |  | *Trifolium pratense* | Tp57577_TGAC_v2_mRNA34847 | Tp57577_TGAC_v2_mRNA34847 |
|  |  | *Vigna unguiculata* | Vigun08g130900.1 | Vigun08g130900 |
|  |  | *Vitis vinifera* | GSVIVT01024622001 | GSVIVT01024622001 |
|  | Monocotyledon | *Ananas comosus* | Aco001607.1 | Aco001607 |
|  |  | *Musa acuminata* | GSMUA_Achr10T01850_001 | GSMUA_Achr10T01850 |
|  |  |  | GSMUA_Achr3T09930_001 | GSMUA_Achr3T09930 |
| Pink | Monocotyledon | *Brachypodium distachyon* | Bradi1g65000.1 | Bradi1g65000 |
|  |  | *Brachypodium hybridum* | Brahy.D01G0892500.1 | Brahy.D01G0892500 |
|  |  |  | Brahy.S02G0156400.1 | Brahy.S02G0156400 |
|  |  | *Brachypodium stacei* | Brast02G149400.1 | Brast02G149400 |
|  |  | *Brachypodium sylvaticum* | Brasy2G179500.1 | Brasy2G179500 |
|  |  | *Hordeum vulgare* | HORVU4Hr1G055040.1 | HORVU4Hr1G055040 |
|  |  | *Miscanthus sinensis* | Misin01G387600.1 | Misin01G387600 |
|  |  |  | Misin02G375900.1 | Misin02G375900 |
|  |  | *Oropetium thomaeum* | Oropetium_20150105_01050A | Oropetium_20150105_01050A |
|  |  | *Oryza sativa* | LOC_Os03g18720.1 | LOC_Os03g18720 |
|  |  | *Panicum hallii* | Pahal.I04382.1 | Pahal.I04382 |
|  |  | *Panicum virgatum* | Pavir.Ia03561.1 | Pavir.Ia03561 |
|  |  |  | Pavir.Ib01390.1 | Pavir.Ib01390 |
|  |  | *Setaria italica* | Seita.9G436100.1 | Seita.9G436100 |
|  |  | *Setaria viridis* | Sevir.9G439900.1 | Sevir.9G439900 |
|  |  | *Sorghum bicolor* | Sobic.001G402300.1 | Sobic.001G402300 |
|  |  | *Spirodela polyrhiza* | Spipo23G0043700 | Spipo23G0043700 |
|  |  | *Triticum aestivum* | Traes_4AS_48684D448.2 | Traes_4AS_48684D448 |
|  |  |  | Traes_4BL_9E4832414.1 | Traes_4BL_9E4832414 |
|  |  |  | Traes_4BL_D59363360.2 | Traes_4BL_D59363360 |
|  |  |  | Traes_4DL_CE62F16B1.1 | Traes_4DL_CE62F16B1 |
|  |  | *Zea mays* | GRMZM2G007590_T01 | GRMZM2G007590 |
|  |  |  | GRMZM2G153450_T01 | GRMZM2G153450 |
|  |  | *Zostera marina* | Zosma141g00120.1 | Zosma141g00120 |
| White | Fern | *Selaginella moellendorffii* | 184747 | 184747 |
| Green | Bryophyte | *Marchantia polymorpha* | Mapoly0092s0049.1 | Mapoly0092s0049 |
|  |  | *Physcomitrella patens* | Pp3c7_10880V3.1 | Pp3c7_10880V3 |
|  |  | *Sphagnum fallax* | Sphfalx0040s0141.1 | Sphfalx0040s0141 |
|  |  |  | Sphfalx0066s0098.1 | Sphfalx0066s0098 |
|  | Monocotyledon | *Ananas comosus* | Aco021518.1 | Aco021518 |
|  |  | *Spirodela polyrhiza* | Spipo3G0033200 | Spipo3G0033200 |
|  |  | *Zostera marina* | Zosma22g01380.1 | Zosma22g01380 |
| Yellow | Algae | *Botryococcus braunii* | Bobra.0203s0006.1 | Bobra.0203s0006 |
|  |  |  | Bobra.0203s0007.1 | Bobra.0203s0007 |
|  |  | *Chlamydomonas reinhardtii* | Cre13.g566700.t1.1 | Cre13.g566700 |
|  |  | *Chromochloris zofingiensis* | Cz11g28070.t1 | Cz11g28070 |
|  |  | *Coccomyxa subellipsoidea* | 14037 | 14037 |
|  |  | *Dunaliella salina* | Dusal.0383s00015.1 | Dusal.0383s00015 |
|  |  | *Micromonas pusilla* | 179440 | 179440 |
|  |  | *Micromonas sp.RCC299* | 107397 | 107397 |
|  |  | *Ostreococcus lucimarinus* | 48819 | 48819 |
|  |  | *Volvox carteri* | Vocar.0001s1517.1 | Vocar.0001s1517 |
