# Supplementary material for: Phylogenetic analysis and stress response of the plant U2 small nuclear ribonucleoprotein B″ gene family
Source: BMC Genomics. 2022 Nov 8;23:744. doi: 10.1186/s12864-022-08956-0 (PMC9644473; doi:10.1186/s12864-022-08956-0)
Supplement: Supplementary file 5 — Additional file 5: Table S4. Expression of U2B″ in rice under different stresses. [file 12864_2022_8956_MOESM5_ESM.docx]

**Table S4 Expression of *U2B”* in rice under different stresses.**

| Position | Treatment time (h) | Drought | Cold | Salt | Cadmium |
| --- | --- | --- | --- | --- | --- |
| Root | 0 | 1.00±0.1 a | 1.01±0.1 a | 1.00±0.07 a | 1.00±0.02 a |
| Root | 3 | 1.06±0.1 a | 0.88±0.07 a | 0.40±0.04 d | 0.92±0.05 a |
| Root | 6 | 1.09±0.1 a | 0.58±0.09 b | 0.85±0.03 b | 0.98±0.1 a |
| Root | 12 | 1.03±0.1 a | 0.92±0.02 a | 0.56±0.03 c | 0.85±0.05 a |
| Shoot | 0 | 1.01±0.1 b | 1.00±0.1 c | 1.00±0.1 ab | 1.01±0.1 a |
| Shoot | 3 | 1.97±0.3 a | 1.41±0.03 a | 0.81±0.08 c | 0.59±0.07 b |
| Shoot | 6 | 2.13±0.6 a | 1.04±0.1 c | 1.11±0.04 a | 0.77±0.09 b |
| Shoot | 12 | 1.57±0.1 c | 1.2±0.04 b | 0.95±0.04 bc | 0.60±0.1 b |

**Different lowercase letters indicate significant differences (P<0.05). At least three biological replicates were performed in all experiments. Statistical analysis was performed using Dunnett's multiple comparisons test by** **GraphPad Prism v9.0.**
