# Supplementary material for: Phylogenetic analysis and stress response of the plant U2 small nuclear ribonucleoprotein B″ gene family
Source: BMC Genomics. 2022 Nov 8;23:744. doi: 10.1186/s12864-022-08956-0 (PMC9644473; doi:10.1186/s12864-022-08956-0)
Supplement: Supplementary file 6 — Additional file 6: Table S5. Sequence summary of plant U2B″ protein-protein interaction network. [file 12864_2022_8956_MOESM6_ESM.docx]

**Table S5 Sequence summary of plant U2B” protein-protein interaction network.**

| Organism | Protein Name | Description | Peptide length | Chr | Start position | End position |
| --- | --- | --- | --- | --- | --- | --- |
| *Arabidopsis thaliana* | AT2G30260 | U2 small nuclear ribonucleoprotein B" | 232 | 2 | 12905402 | 12907509 |
|  | AT1G20580 | Small nuclear ribonucleoprotein family protein | 131 | 1 | 7128884 | 7130642 |
|  | F17A22.3 | Small nuclear ribonucleoprotein family protein | 109 | 3 | 23235490 | 23236816 |
|  | Y14 | RNA-binding (RRM/RBD/RNP motifs) family protein | 202 | 1 | 19102799 | 19104850 |
|  | AT5G44500 | Small nuclear ribonucleoprotein family protein | 254 | 5 | 17927453 | 17929463 |
|  | AT4G30330 | Small nuclear ribonucleoprotein family protein | 88 | 4 | 14836548 | 14838020 |
|  | smB | Small nuclear ribonucleoprotein associated protein B | 257 | 4 | 11022142 | 11023752 |
|  | AT5G64270 | Splicing factor, putative | 1269 | 5 | 25706659 | 25710925 |
|  | U2A | U2 small nuclear ribonucleoprotein A | 249 | 1 | 3159125 | 3161692 |
|  | AT2G18740 | Small nuclear ribonucleoprotein family protein | 88 | 2 | 8123221 | 8125004 |
| *Oryza sativa* | LOC_Os03g18720 | U2 small nuclear ribonucleoprotein B" | 232 | 3 | 10478135 | 10482626 |
|  | OsJ_06008 | U2 small nuclear ribonucleoprotein A | 284 | 2 | 7630852 | 7635234 |
|  | OS08T0154700-01 | Sm-like protein LSM2 | 93 | 8 | 3145064 | 3148942 |
|  | OsJ_18021 | Putative small nuclear ribonucleoprotein D2 | 105 | 5 | 14498020 | 14500793 |
|  | OsJ_34703 | Small nuclear ribonucleoprotein F | 86 | 11 | 27025613 | 27027847 |
|  | OsJ_05002 | Putative small nuclear ribonucleoprotein D3 | 132 | 2 | 141146 | 143941 |
|  | OsJ_07305 | Putative small nuclear ribonucleoprotein D1 | 114 | 2 | 23041495 | 23044960 |
|  | OsJ_10232 | Splicing factor 3A subunit 2 | 321 | 3 | 8786802 | 8789721 |
|  | OsJ_05214 | Putative small nuclear ribonucleoprotein E | 88 | 2 | 1399613 | 1401222 |
|  | OS03T0240800-01 | Small nuclear ribonucleoprotein D3 | 132 | 3 | 7449782 | 7453038 |
|  | OS08T0151400-01 | Small nuclear ribonucleoprotein E | 64 | 2 | 1422022 | 1424096 |
| *Zea mays* | GRMZM2G007590 | U2 small nuclear ribonucleoprotein B" | 233 | 9 | 137157996 | 137166081 |
|  | IDP2400 | U2 small nuclear ribonucleoprotein A | 284 | 1 | 298511319 | 298515696 |
|  | 103649582 | Small nuclear ribonucleoprotein E | 177 | 3 | 3504545 | 3508163 |
|  | Ay110400 | Sm-like protein LSM8 | 99 | 6 | 162862269 | 162867072 |
|  | GRMZM2G413193_P01 | U1 small nuclear ribonucleoprotein C | 421 | 4 | 224410980 | 224416043 |
|  | GRMZM2G494600_P01 | Unknown | 487 | 5 | 204431009 | 204437995 |
|  | GRMZM5G884325_P01 | Small nuclear ribonucleoprotein D3 | 132 | 1 | 32326773 | 32333363 |
|  | GRMZM2G014091_P01 | Small nuclear ribonucleoprotein E | 88 | 8 | 24416410 | 24435978 |
|  | 100284316 | Small nuclear ribonucleoprotein D3 | 132 | 9 | 143864968 | 143869347 |
|  | C3H42 | C2H2 and C2HC zinc fingers superfamily protein | 166 | 8 | 5908910 | 5912846 |
|  | pco138115 | Small nuclear ribonucleoprotein D3 | 132 | 5 | 66868787 | 66871798 |
